# Explainable AI for gastrointestinal lesion surveillance and precision targeted drug delivery

**DOI:** 10.1038/s41598-026-40882-z

**Published:** 2026-03-23

**Authors:** Islam R. Kamal, S. F. El-Zoghdy, Randa F. Soliman

**Affiliations:** 1 Department of Computer Science, Faculty of Information Systems and Computer Science, October 6 University, Giza, 12585 Egypt; 2 Department of Mathematics and Computer Science, Faculty of Science, Menoufia University, Shebin El-Kom, 32511 Egypt; 3 Machine Intelligence Department, Faculty of Artificial Intelligence, Menoufia University, Shebin El-Kom, 32511 Egypt

**Keywords:** Internet of bio-nanothings, Healthcare applications, Nano-sensors, Wireless ingestible imaging devices, Quadratic map privacy algorithm, Nanotechnology, Explainable AI, Engineering, Mathematics and computing, Nanoscience and technology

## Abstract

The Internet of Bio-NanoThings (IoBNT) promises revolutionary healthcare applications, particularly in targeted drug delivery. However, major challenges remain including safe nanodevice design, monitoring their behavior in biological environments and enabling reliable communication with external control systems. This work proposes an AI-assisted IoBNT architecture that combines gastrointestinal (GI) imaging with intelligent therapeutic supervision. A wireless ingestible imaging device (WIID) captures GI images, while an Artificial Intelligence Ciphered Link (AICL) analyzes them using convolutional neural networks (CNNs) trained with supervised contrastive learning and cost-sensitive fine-tuning. Unlike prior studies focused solely on tumors, our system is evaluated on the HyperKvasir dataset covering 25 GI disease classes, including neoplastic and inflammatory conditions. Explainable AI methods (GradCAM family) are employed with quantitative validation to improve model transparency. Drug transport and release are modeled using a multi-compartment pharmacokinetic framework with uncertainty analysis. Security protections using Quadratic Map Privacy Algorithm (QMPA), threat modeling and failsafe dosing limits are incorporated to enhance clinical safety. The system achieves 91.4% classification accuracy (weighted F1 = 0.91) on HyperKvasir, with stronger performance in neoplastic classes and lower accuracy in rare categories, emphasizing the importance of class-balanced evaluation. These results demonstrate the feasibility of integrating AI-based disease detection with controlled drug delivery, representing a step toward closed-loop, adaptive IoBNT therapeutics.

## Introduction

The field of nanotechnology has revolutionized scientific progress through the development of novel methodologies, tools, and systems that enhance conventional technologies while facilitating unprecedented scientific breakthroughs^1^. Through precise manipulation of materials at molecular and nanoparticle dimensions, this field enables the development of biologically compatible microscale systems called bio-nanomachines, which possess the capability to interface with living systems across cellular and whole-organism scales. These bio-nanomachines demonstrate diverse capabilities, as outlined in^2^, including the identification, isolation, preservation, discharge, and integration of biomolecules, alongside functions such as energy production and utilization, movement generation, object manipulation and the regulation of biological activities. Despite doxorubicin (Dox) established effectiveness as a chemotherapeutic agent for various malignancies, clinical application faces constraints due to dose-dependent cardiac toxicity that limits total lifetime exposure. These cardiotoxic risks, linked to drug plasma levels, have driven investigations into refined dosing strategies aimed at preserving therapeutic effectiveness against tumors while reducing cardiac adverse effects^3^. Within this context, nanotechnology offers nanoparticle (NP)-based drug carriers capable of delivering and releasing medication specifically at tumor sites, thereby improving therapeutic precision. In addition, nanotechnology enables the use of molecular communication (MC) to interconnect bio-nanomachines. The importance of MC is its ability to advance the understanding of complex diseases (e.g., malignant cell behavior) by identifying key intracellular and extracellular processes within bio-nanomachine networks. These processes can be effectively managed through externally controlled systems. The Internet of Bio-NanoThings (IoBNT)^4–8^, which integrates molecular communication with AI-based ciphered links, represents a promising approach for bridging internal MC environments with external networks such as the Internet.

MC holds significant promise for nanomedicine applications, particularly in targeted drug delivery systems (TDDS), which remain a major focus of current research^9–11^. The leading objective of TDDS is to deliver a therapeutic agent directly to the target site while minimizing adverse effects on healthy tissues^12^. In an MC-based TDDS, the transmitter nanomachine carries and releases the drug, with drug molecules serving as the information carriers. The blood plasma functions as the communication channel, while the targeted or tumor tissue acts as the receiver nanomachine^9,10^.

Designing and implementing a functional IoBNT has diverse critical challenges. These encounter the development of advanced artificial nano-devices, the establishment of reliable molecular communication frameworks within nano-networks, and the creation of effective interfaces between nano-networks and the Internet. Previous IoBNT research has primarily focused on optimizing communication in artificial nanomachines within certain nano-networks. This is particularly important in healthcare applications, where signal integrity ensures that drug particles remain separated within the vascular system until they reach their intended targets. However, many existing studies tend to neglect potential bad effects on healthy cells and the prolonged circulation of drugs in the bloodstream. In addition, certain medical conditions, such as aggressive and localized cancers, require highly precise treatment approaches, making accurate tumor localization critical, failure in this regard can lead to serious consequences. Further research is also needed on rate-control mechanisms to regulate particles information liberation based on environmental situations^13,14^. As discussed earlier, IoBNT still faces several challenges, including model security, controlling and monitoring nano-carrier diffusion within the body nanonetwork (BAN) and establishing effective interfaces between the nanonetwork and the Internet. A primary challenge is the ability to monitor and track nanodevices near infected or diseased areas. To address these issues, this paper explores IoBNT and its medical applications in healthcare surveillance and targeted drug delivery. We present an IoBNT-based monitoring and drug delivery model employing two distinct approaches.

First, we introduce wireless ingestible imaging device (WIID), a novel diagnostic and monitoring technique that offers a relatively painless and non-invasive alternative to traditional methods^15–18^. WIID are resistant to stomach enzymes, capable of traversing vascular barriers, and designed to visualize entire target tissues. Each device is extremely small and equipped with a battery, transmitter, LED light source, and color video camera. As the device moves through tissue, the camera automatically captures images, which are transmitted via an onboard wireless transmitter to a body-mounted receiver (worm sensor).

Second, we propose an intelligent control layer, the AI Ciphered Link (AICL), which operates atop a Multi-Compartmental Pharmacokinetic Model (MCM) to enable precise, closed-loop therapeutic intervention. The AICL leverages Convolutional Neural Networks (CNNs) for real-time gastrointestinal (GI) pathology detection and classification, processing the video stream from WIID. To ensure robustness against severe class imbalance, we employ a two-stage training strategy: an encoder is first trained using supervised contrastive learning^19^ to generate discriminative image embeddings, followed by cost-sensitive fine-tuning of a classification head that prioritizes clinical severity. We rigorously benchmark state-of-the-art architectures (e.g., EfficientNet, MobileNetV3) across 25 GI classes, while transparently acknowledging the dataset’s broad scope beyond oncology to reflect real-world clinical complexity. To foster trust and clinical utility, we integrate Explainable AI (XAI) techniques including GradCAM, GradCAM +  + , ScoreCAM, and LayerCAM and validate them quantitatively. Our design directly addresses critical deployment barriers through rigorous security threat modeling, formal safety-constrained control policies, and comprehensive pharmacokinetic uncertainty analysis, ensuring a clinically translatable IoBNT system.

The main contributions of current study can be summarized as follows:

- Development of an AICL device capable of precise GI detection and classification, while simultaneously enabling bidirectional control and transfer of therapeutic drug doses.
- Introduction of a secure, end-to-end therapeutic delivery architecture grounded in multi-compartmental pharmacokinetic modeling with comprehensive parameter uncertainty analysis (Monte Carlo simulation, n = 10,000; sensitivity analysis; 90% prediction intervals), ensuring robust therapeutic outcomes across physiological variability.
- Formal specification of closed-loop control policies with explicit safety constraints (rate limiting, maximum dose bounds, failsafe defaults, manual override), and quantitative XAI validation ensuring reliable clinical decision support.
- Security threat modeling and mitigation strategies for sensitive medical data and therapeutic commands, including encryption, authentication, hardware interlocks, and regulatory compliance considerations (HIPAA, FDA).
- Comprehensive evaluation of the proposed framework to demonstrate its effectiveness, reliability and potential for improving precision in tumor diagnosis and treatment.

The organization of this paper is as follows: Section "Related work" reviews the related work. Section "Proposed framework" presents the details of the proposed framework. In Section "The artificial intelligence ciphered link framework", the AICL framework is described, and Section "The proposed multi compartmental framework using ciphered link" introduces the proposed model. Section "Simulation results and discussions" reports and discusses the numerical results. Finally, Section “Conclusion” concludes the paper by summarizing the key findings and outlining potential future directions.

## Related work

Integrating health monitoring via implantable medical devices, referred to as Target Drug Delivery systems (TDDs), with Information and Communication Technology (ICT) through the Internet of Bio-Nano Things (IoBNT) represents a significant advancement in healthcare technology. As outlined in^11^, the IoBNT framework demonstrates considerable potential for enhancing healthcare delivery. A key feature of this framework is the biocyber interface, which bridges biochemical signaling-based bio-nano networks with the electromagnetic-based Internet. However, a major challenge in implementing IoBNT lies in achieving precise and reliable drug delivery to targeted cells, an area where conventional methods often prove inadequate. To address this limitation, the proposed model introduces novel parameters designed to enhance the efficiency and reliability of therapeutic drug delivery. Specifically, the analysis highlights the cooperative role of the bio-cyber interface and the vascular diffusion network in directing therapeutic agents to their intended intra-body nano-network targets.

In this context^9^, introduces an MC approach for TDD, capable of delivering therapeutic agents to multiple targeted sites. This method utilizes nano-transmitters and nano-receivers implanted within a designated vascular network, enabling drug molecules to be transported into infected regions via a Compartmental Model (CM). The CM facilitates targeted delivery by allowing therapeutic molecules to diffuse toward the surface of diseased tissues, thereby improving treatment efficacy. The success of such systems, however, depends on several critical factors, including the size of the nano-receiver, the diffusion rate, the total volume capacity of the nano-transmitters, and the concentration of enzymes present in the bloodstream.

The authors of^12^ introduced an advanced Dox drug delivery system that enhances the therapeutic window by improving both safety and efficacy compared to existing FDA-approved versions. However, their approach does not utilize IoBNT for remote guidance of drug particles. In a complementary study, reference^3^ developed an analytical model of Dox cytotoxicity. This model, based on a sigmoidal Hill-type response, shows that cell death is dependent on both intracellular and extracellular drug concentrations. By quantifying cumulative damage based on peak drug levels in both environments, the study highlights that precise dosage control is essential for maximizing therapeutic benefit while minimizing side effects. The potential of the IoBNT and MC to advance TDD is well-documented. Several works provide a broad overview, discussing the technical foundations of these fields and their applications in biomedical monitoring, precision healthcare, and oncology^13,15,16^. Beyond these reviews, specific innovative systems are being developed. For example^14^, introduced a bio-inspired MC system that utilizes a multi-input multi-output (MIMO) Förster Resonance Energy Transfer (FRET) nano relay to transport drugs from extravascular to intravascular regions. In another advancement^17^, proposed a novel bio-nano-thing (BNT) architecture called Molecular Nano Neural Networks (M3N), which enables network-based intelligence at the micro- and nano-scales by interconnecting simple functional compartments.

While these works provide essential theory for diffusion, channel characterization and nano-device networking, they operate under a critical simplifying assumption: the therapeutic target is predefined and static. These models lack a mechanism for real-time, sensing-driven target identification or adaptive control separating them from a responsive, closed-loop therapeutic system. Concurrently, major advances have occurred in automated GI pathology detection using deep learning on endoscopic imagery, as evidenced by benchmarks like the HyperKvasir dataset. CNNs now achieve high diagnostic accuracy for conditions ranging from neoplasia to inflammation. However, the focus of this field is almost exclusively diagnostic and classificatory. State of the art AI models function as sophisticated pattern recognizers but are not designed to interface with or control therapeutic actuators. Their output is a diagnostic label not a calibrated actionable command for a drug delivery system. This constitutes a termination point in the clinical workflow rather than the initiation point of a targeted therapy.

A third critical domain involves computational modeling of drug transport and effect. This includes analytical models of cytotoxicity, such as sigmoidal Hill-type responses linking cell death to intra- and extracellular drug concentrations^3^, and advanced simulations that incorporate tumor microenvironment (TME) factors like ECM density, pH and hypoxia^18–20^. Integrating PK/PD frameworks allows for dynamic characterization of drug distribution and therapeutic outcome. While these models offer profound biological insight and predictive power, they are predominantly deployed as offline simulation and analysis tools. They are not architected as real-time state estimators or controllers within an operational IoBNT feedback loop.

A review of these parallel tracks reveals a fundamental disconnect. MC and nano-network research provides the “delivery vehicle” but lacks the “driver” (intelligent, sensor-driven guidance). AI imaging provides the “eyes” (diagnosis) but lacks the “hands” (therapeutic action). PK/PD-TME models provide the “map” of biological dynamics but are not connected to the “steering wheel” of real-time control. Consequently, no existing framework jointly integrates (i) medical image understanding, (ii) supervised AI decision-making, (iii) multi-compartment drug propagation modeling and (iv) closed-loop therapeutic control within a unified, security-aware IoBNT architecture. Furthermore, practical requisites for clinical translation such as rigorous explainability validation, comprehensive parameter uncertainty analysis (e.g., via large-scale Monte Carlo simulation), cybersecurity for command authentication, and safety-bounded dosing policies are often secondary or absent in prior art. This work directly addresses this integration gap. We present an IoBNT system architecture that establishes a security-aware, explainable and safety-constrained closed loop. It formally links AI-based GI pathology classification (validated on diverse clinical data) to adaptive drug release via a multi-compartmental pharmacokinetic model, with all components subjected to rigorous uncertainty quantification and failsafe analysis, thereby bridging the divide between diagnostic AI, communication theory and translational therapeutic design.

## Proposed framework

**Fig. 1:**
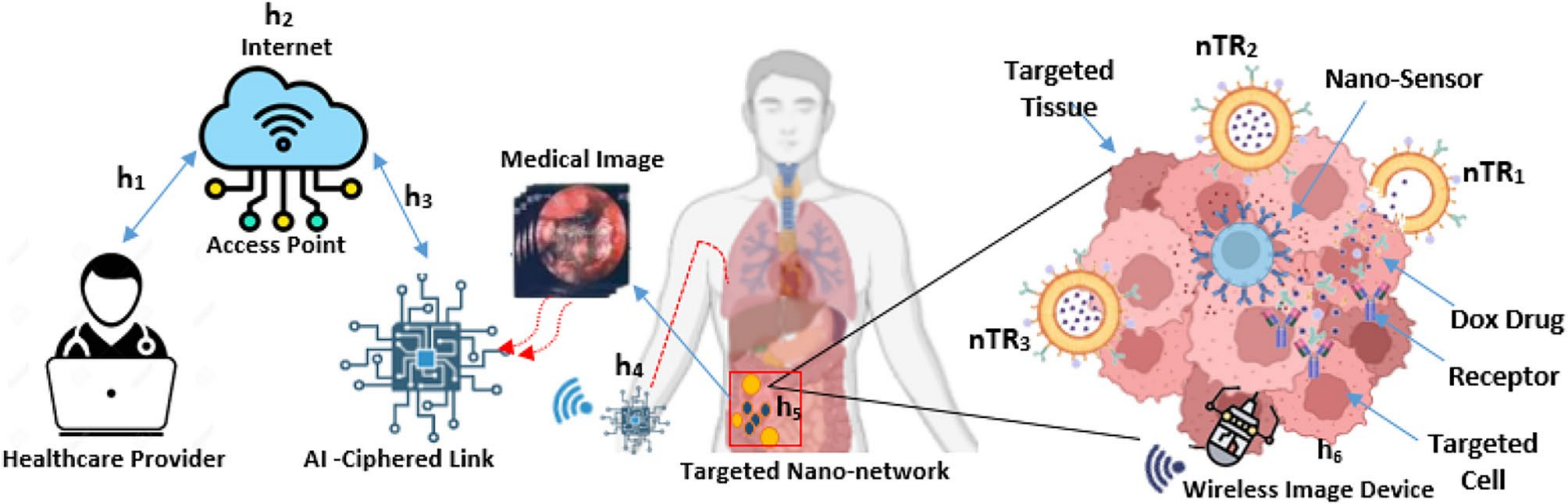
IoBNT-based wireless ingestible imaging devices layout.

The framework proposed in this work is depicted in Fig. 1. It comprises seven main elements: the Internet, an access point, a wireless channel, an AICL device, the blood vessel channel, and a WIID located in the target tissue. Drawing an analogy from communication systems^6^, each component is modeled as a time-varying impulse response. The system operates when medical staff transmit binary instructions online to manage functions like drug particle release, therapeutic agent synthesis, and the diffusion of nano-carriers.

This paper focuses on modeling three specific modules. The first module covers communication from the WIID to the AICL, where a pre-trained model is applied for tumor cell classification and detection. The second module involves data transmission through the wireless channel, linking the AICL device to the bloodstream tubing. The third module addresses communication from the bionanosensor to medical specialists via the blood vessel. In this stage, biochemical signals detected within the bloodstream are transformed into electrical signals by the nanosensor. These signals, derived from bioluminescence reactions, are then transmitted by the AICL device to medical personnel over the Internet.

The model design objectives are twofold: (1) to establish a ciphered link using CNN-based strategies that support specialists in making timely and informed clinical decisions, and (2) to achieve therapeutic effectiveness by localizing drug action within targeted cells, thereby slowing or halting disease progression. In addition, the framework seeks to overcome critical challenges such as rapid drug absorption, dose clearance, and the reduction of side effects in non-targeted tissues.

At this stage, it is essential to outline the functioning of our system, beginning with the specialist and concluding with the delivery of the therapeutic dose to the targeted tumor cells. The specialist initiates the process by transmitting command information through the access point. Subsequently, the connection among ${\boldsymbol{h}}_{1}$ **(t),** ${\boldsymbol{h}}_{2}$ **(t)** and $\user2{ h}_{3}$ **(t**) can be represented as a wireless channel, expressed as follows

1 $$z^{n} \left( t \right) = y^{\left( n \right)} \left( t \right)*\left( {h_{1} \left( t \right)*h_{2} \left( t \right)*h_{3} \left( t \right)} \right), \, n = \{ f, \, r\} .$$

In Eq. (1), $\user2{ y}^{{\left( {\boldsymbol{n}} \right)}}$ denotes the input signal, and * represents the convolution operator. The variable ***n*** specifies the communication path: ***f*** ndicates forward communication, while ***r*** denotes reverse communication.

Figure 1 illustrates the functional modules of the IoBNT described in^20^, which are integrated into our proposed healthcare delivery system. In this system, doxorubicin (DOX) is delivered to malignant cells within specific tissues. The targeted nano-network encounters of therapeutic artificial machines (**n** ${\boldsymbol{TR}}_{1}$, **n** ${\boldsymbol{TR}}_{2}$, **n** ${\boldsymbol{TR}}_{3}$, WIID and nano-sensor) all of which interface with the intra-body area network. A smart pump, described in^21–23^, regulates the delivery of DOX-loaded artificial machines or direct drug doses. The transmission and dispersion of DOX are facilitated through molecular communication techniques, as discussed in^10,11^. The WIID is responsible for monitoring and tracking nano-devices in the infected region, while the nano-sensor detects chemical signals within the targeted nano-network^24^. Emulating biological cells, the therapeutic artificial machines are engineered with receptors that bind to specific ligands (drugs). This selective binding event serves as the fundamental mechanism for reception. Moreover, these machines function as transceivers, capable of both transmitting and receiving data. Specifically, **n** ${\boldsymbol{TR}}_{{1\user2{ }}}$ is assumed to act as the transceiver machine (similar to PEGylated liposomes), encapsulate the drug (e.g., DOX) and releasing it in response to external stimulus signals. Meanwhile, **n** ${\boldsymbol{TR}}_{2} \user2{ }$ and **n** ${\boldsymbol{TR}}_{3}$ are designed to synthesize and release targeted molecules, thereby supporting and enhancing the overall drug delivery process.

This paper examines two distinct frameworks for delivering drug molecules to diseased cells at targeted sites. The first framework is an external body system that connects medical personnel to an AICL. In this setup, an AI model classifies medical images transmitted through the AICL. Based on the analysis, the system sends encryption signals that instruct bio-nanosensors to release drug-loaded liposomes at the targeted site. The second framework is an internal intra-body nanonetwork, in which the distribution of drug molecules is guided by a multi-compartmental model^25^, incorporating the extracellular channel, conceptual reception^26^, and the spatial relationship between transmitter and receiver. This model facilitates the effective transport of drug molecules within the body, allowing them to reach diseased cells with higher precision. By combining these two frameworks into a dual-channel approach, the proposed system ensures more accurate and controlled delivery of therapeutic agents, improving treatment efficacy while reducing side effects.

## The artificial intelligence ciphered link framework

**Fig. 2:**
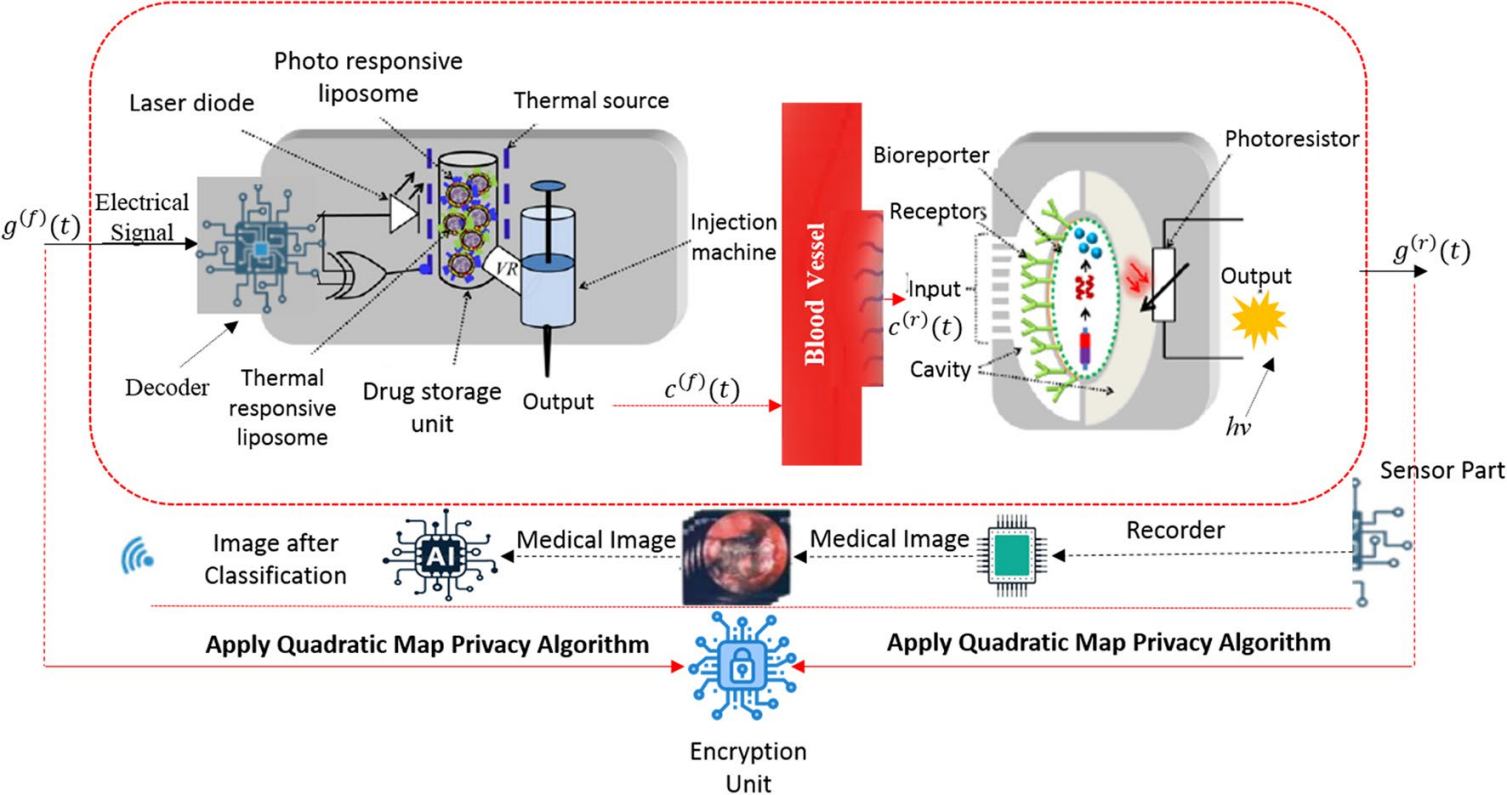
The developed dual AI ciphered link model.

Our proposed architecture centers on a biocyber interfacing mechanism. This configuration incorporates a dual-transduction module that functions by initially receiving control instructions via electromagnetic (EM) wave transmission, subsequently transforming these instructions to activate a transducer component responsible for producing biochemical signaling outputs, as depicted in Fig. 2. The developed biocyber platform operates using ${c}^{(f)}$ (*t*) and ${c}^{(r)}$ (*t*), which denote the electrical and biological signal pathways for forward and reverse transmission modes, respectively. The constructed biocyber interfacing system facilitates bidirectional transformation between electrical and biological signal modalities through the implementation of logic gate circuitry^13^. A CNN model has been integrated within this research to augment both the operational capabilities and overall efficiency of the biocyber interfacing platform.

### The proposed AI ciphered link

**Fig. 3:**
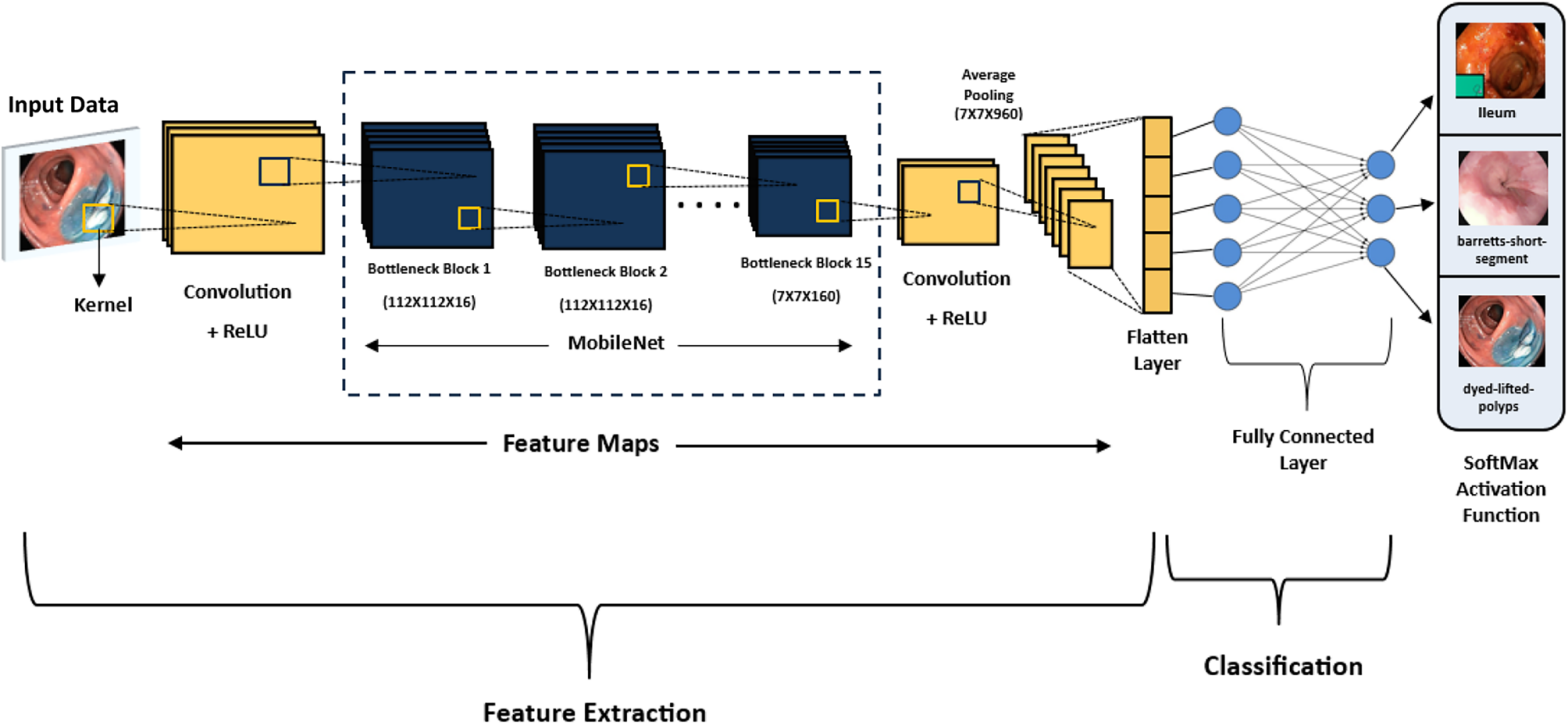
Schematic diagram of the proposed methodology.

The functions of the AI ciphered link are carried out through a sequence of steps in the backward path. First, the WIID naturally travels through the targeted tissue, capturing images of the diseased cells. To demonstrate deployment feasibility under the high image volumes generated per procedure, we define a quantitative deployment profile covering system architecture, throughput, compression, inference partitioning, runtime and clinical workflow integration. The system follows a three-tier hierarchy in which the ingestible WIID captures 320 × 320 RGB images at 2–3 fps using a CMOS sensor with LED illumination, acquiring approximately 50,000–60,000 frames during an 8-h procedure with 16 GB onboard storage and MICS-band wireless transmission, while an AICL wearable (NVIDIA Jetson Nano, 4 GB RAM, 128 CUDA cores) performs real-time edge inference and a hospital workstation conducts post-procedure analysis. At an average 2.5 fps, raw data generation reaches 768 kB/s (3.3 GB/hour), which would exceed storage limits; therefore, adaptive JPEG compression (quality factor 85) is applied, achieving ~ 10:1 compression, reducing storage to 2.9 GB per procedure with only 1.2% absolute accuracy degradation, whereas higher compression ratios caused substantial diagnostic loss. Inference is partitioned hierarchically: the AICL executes INT8-quantized MobileNetV3-Large for binary abnormality screening with < 100 ms latency per frame enabling immediate alerts, while full-precision 25-class classification with temporal analysis and XAI visualization is performed post-procedure (< 30 min), balancing latency, privacy and computational load. The per-frame runtime pipeline capture (0.5 ms), preprocessing (2 ms), compression (8 ms), inference (35 ms), encryption (5 ms) and transmission (50 ms) totals ~ 100 ms, supporting up to 10 fps effective throughput, well above acquisition rate. Resource profiling shows < 25% storage utilization, ~ 30% average CPU load and battery consumption within operational margins, with buffered frame management and intermittent offloading. Clinically, the workflow mirrors standard capsule endoscopy: ingestion, unattended real-time monitoring during daily activity, automated post-procedure download (~ 5 min), AI-assisted analysis (~ 30 min), and radiologist review with XAI outputs confirming that storage, compute, power and timing constraints are compatible with existing hardware and established gastroenterology practice. These images are then transmitted wirelessly to a data recorder on the AICL, which is worn by the patient. The recorder, equipped with sensors, detects and stores the images during the procedure. Once complete, the recorded images are downloaded into specialized software within the ciphered link. At this stage, a CNN is applied for accurate tumor detection and classification. The analysis results are relayed to medical personnel and also to the AICL forward path, where they are used to regulate and control drug delivery in both forward and reverse directions, minimizing the impact on healthy cells. In this study, a pre-trained CNN model (MobileNetV3-Large) is employed to implement the methodology through a structured series of steps. Figure 3 presents a schematic overview of the proposed approach, with the subsequent sections detailing each step in the process.

#### Step-by-step methodology

**Step 1 – Data augmentation:**

To increase the diversity of the training data and improve model robustness, we applied several augmentation techniques, including color jittering, color dropping, flipping, and random cropping. These transformations introduced variability into the dataset, allowing the model to learn more generalized representations.

**Step 2 – Image processing:**

All images were resized to a resolution of 224 × 224 to ensure uniformity across the dataset. Standardizing the image dimensions provided consistency and compatibility for subsequent processing, enabling efficient analysis in later stages of the workflow.

**Step 3 – Contrastive training:**

Our method uses a supervised contrastive learning framework to learn discriminative feature representations. The encoder is built on a pre-trained MobileNetV3-Large backbone, denoted as *f*(·). To project these features into an embedding space suitable for contrastive loss, we attach a projection head *g*(·). This head comprises two fully connected layers (512 → 128 units) with ReLU activation and L2 regularization, outputting L2-normalized embeddings. The entire system is trained end-to-end with a MaxMargin contrastive loss, an adaptation of the SimCLR principle that leverages label information to explicitly enforce inter-class separation during training. Given a mini-batch of (*N*) images with labels, the supervised contrastive loss is defined as

2 $$L_{SupCon} = \mathop \sum \limits_{i = 1}^{N} \frac{ - 1}{{\left| {P\left( i \right)} \right|}} \mathop \sum \limits_{p \in P\left( i \right)} log\frac{{{\mathrm{exp}}\left( {z_{i } . z_{p} /T} \right)}}{{\mathop \sum \nolimits_{a \in A\left( i \right)} {\mathrm{exp}}\left( {z_{i } . z_{p} /T} \right)}}$$

where ${z}_{i }=\frac{g(f\left({x}_{i }\right))}{|\left|g\left(f\left({x}_{i }\right)\right)\right||}$ is the L2-normalized embedding, $P\left(i\right)$ is the set of **positive samples** sharing the same class label, $A\left(i\right)$ contains all samples in the batch except and *T* is a temperature parameter (set to 0.07).

Positive pairs are constructed from images belonging to the same pathology class, while negative pairs correspond to images from different classes. This encourages intra-class compactness and inter-class separability in the embedding space.

**Step 4 – Classifier with frozen encoder:**

The encoder extracted feature vectors from input images, which were then passed to a classifier consisting of a fully connected layer and a softmax layer. During fine-tuning, the encoder weights were frozen. To address severe class imbalance (class support ranging from 5 to 2000 samples), we implemented **Focal Loss**^27^ with *γ* = 2 to down-weight easy majority-class examples, combined with **class-balanced batch sampling** ensuring minimum 4 samples per class per batch. Class weights were computed as *w*_*c*_ = *n*_*samples*_/(*n*_*classes*_* n*_*c*_). The classifier was trained for 100 epochs using this composite loss function.

**Step 5 – Interpretation with explainable AI (XAI) and quantitative validation**

To enhance transparency, we applied GradCAM, GradCAM +  + , Faster ScoreCAM, and LayerCAM^28^. However, CAM-based methods have known limitations in medical imaging: (i) sensitivity to noise and adversarial perturbations, (ii) tendency to highlight discriminative but non-causal regions, (iii) dependence on final convolutional features that may miss fine pathological details. To validate explanation fidelity, we implemented:

**A.** Deletion Metric (Faithfulness): Measure prediction drop when removing highlighted regions. For explanation map $$M$$ and image $$I$$, define: $$Del\left(AUC\right)={\sum }_{i=1}^{N}|f\left(I\right)-f(I$$ ⊙ (1-$${M}_{i}))|$$, where $${M}_{i}$$ is binary mask at top-$$i$$% threshold. Lower deletion score indicates better localization.

**B.** Insertion Metric: Measure prediction rise when adding highlighted regions to uniform background. Higher insertion score indicates better localization.

**C.** Stability: Compute Intersection-over-Union (IoU) between explanations from 5 independent training seeds. IoU > 0.7 indicates stable, reproducible explanations.

**D.** Ground truth overlap: For 100 randomly selected images with expert-annotated lesion boundaries, compute Pixel-wise IoU between CAM activation (thresholded at 50% max) and ground truth mask. This approach not only validated the model’s reliability but also improved understanding of its classification behavior.

**Fig. 4:**
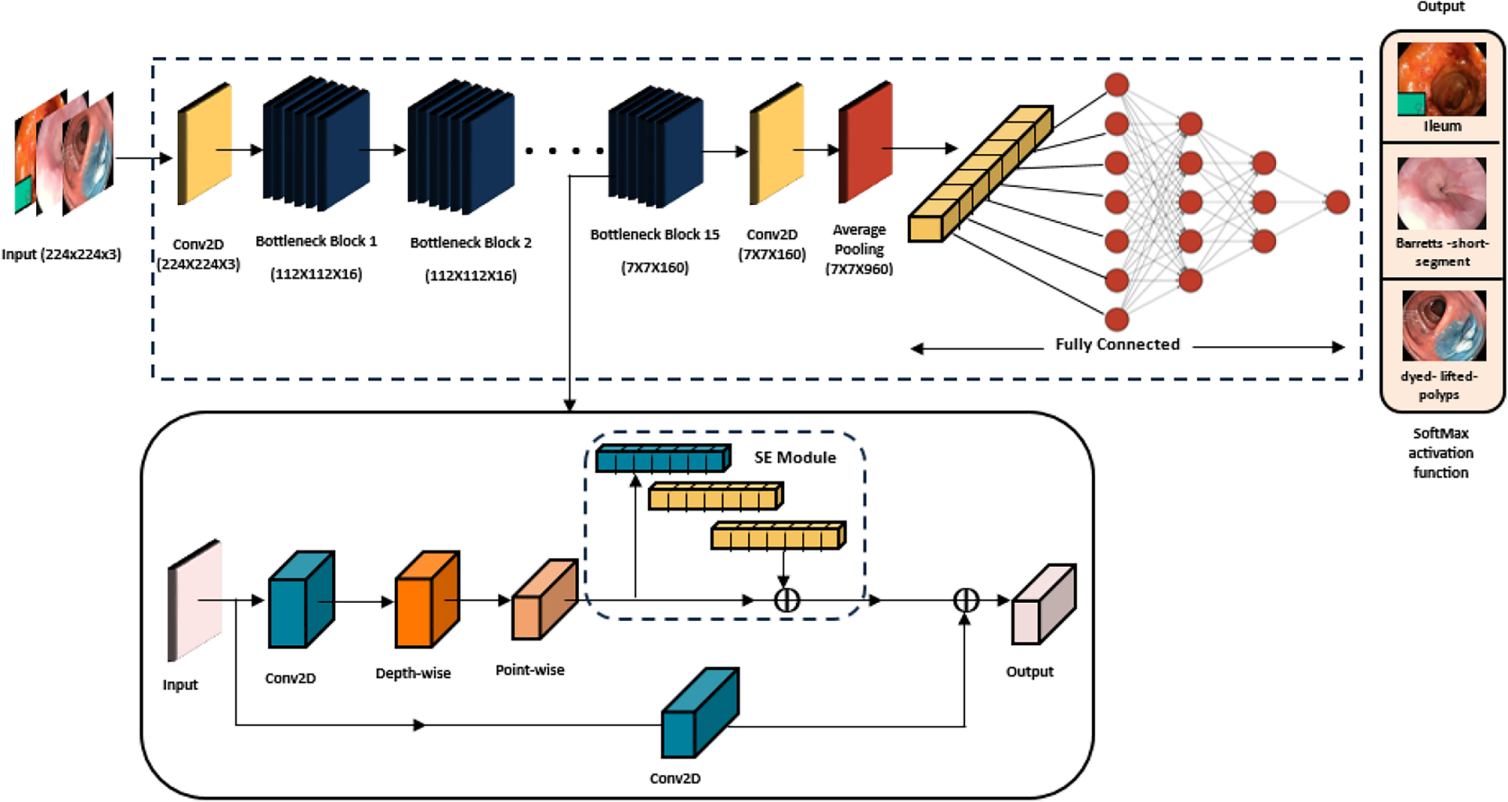
CNN using MobileNet V3Large architecture diagram.

Figure 4 illustrates the initial steps of our model architecture. The process begins with standard convolution, which adds dimensionality for sequential bottlenecks. Next, the network applies batch normalization, which both normalizes activation values to stabilize training and introduces non-linear capabilities that enhance feature extraction from the affected regions.

This module employs a series of bottleneck blocks to extract discriminative features. Each bottleneck block includes an expansion step to increase the input dimensionality before applying the linear activation function. Depthwise-separable convolution is then used sequentially to capture subtype features, resulting in substantial computational savings, improved efficiency, and reduced processing time. In addition, each bottleneck incorporates a squeeze-and-excitation (SE) module, which improves the network’s ability to focus on the most relevant regions. The hyperparameters of each bottleneck block are detailed in Table 1. After feature extraction, the outputs are aggregated using a mean pooling layer and then passed to a fully connected layer to generate fundamental feature representations. Finally, the softmax activation function is applied to produce the class predictions. MobileNet architecture integrates three key components: the linear bottleneck, the SE module, and depthwise-separable convolution. Among these, the depthwise-separable convolution plays a central role in achieving high efficiency without compromising accuracy. Unlike standard convolution, it separates the process into two stages: depthwise convolution, which applies a single filter to each input channel, and pointwise convolution, which applies a 1 × 1 convolution to combine the outputs. This factorization greatly reduces computation compared to standard convolution. Furthermore, the inclusion of SE modules improves feature representation by leveraging attention mechanisms to reweight channel responses. This significantly enhances the network’s ability to extract meaningful features while maintaining computational efficiency.

**Table 1:** Specification for MobileNetV3-Large.

Operator	Kernel size	Exp size	#Out	SE	NL	S
Conv2D	3 × 3	–	16	–	HS	2
Bottleneck	3 × 3	16	16	–	RE	1
Bottleneck	3 × 3	64	24	–	RE	2
Bottleneck	3 × 3	72	24	–	RE	1
Bottleneck	5 × 5	72	40	✓	RE	2
Bottleneck	5 × 5	120	40	✓	RE	1
Bottleneck	5 × 5	120	40	✓	RE	1
Bottleneck	3 × 3	240	80	–	HS	2
Bottleneck	3 × 3	200	80	–	HS	1
Bottleneck	3 × 3	184	80	–	HS	1
Bottleneck	3 × 3	184	80	–	HS	1
Bottleneck	3 × 3	480	112	✓	HS	1
Bottleneck	3 × 3	672	112	✓	HS	1
Bottleneck	5 × 5	672	160	✓	HS	2
Bottleneck	5 × 5	960	160	✓	HS	1
Bottleneck	5 × 5	960	160	✓	HS	1
Conv2D	1 × 1	–	960	–	HS	1

**SE** denotes whether there is a squeeze-and excite in that block. **NL** denotes the type of nonlinearity used. Here, **HS** denotes h-swish and **RE** denotes ReLU. **S** denotes stride.

### AI-guided closed-loop drug release control policy

This section presents a complete mathematical and systems-level specification of the proposed closed-loop therapeutic controller. The architecture formalizes the translation of imaging-based diagnostic inferences into adaptive pharmacokinetic (PK) parameter modulation, ensuring robust and safety-constrained operation, which is done through a series of stages.

- Stage1, Control pipeline architecture: The end-to-end control pipeline is formally defined as a deterministic mapping from raw imaging data to therapeutic actuator commands:
  $$\tau \left( t \right)\to ^{CNN} Pc\left( t \right)\to ^{C} u\left( t \right)\to ^{M} \left( {\omega_{0} \left( t \right), \delta \left( t \right),R_{IN} \left( t \right)} \right)$$

where $\tau \left(t\right)$ denotes the multimodal imaging input at time *t*,$Pc\left(t\right)$ ∈ [0, 1] is the neural network-derived confidence score for the pathological class, C represents the discrete control law, *u*(*t*) is the discrete control signal, M is the parameter mapping function, ${\omega }_{0}\left(t\right),$ *δ*(*t*) and ${R}_{IN}\left(t\right)$ are the modulated PK parameters governing drug release rate, release profile sharpness, and infusion duration, respectively.

- Stage2, Discrete supervisory control law: To convert the continuous confidence measure *pc*(*t*) into deterministic therapeutic actions, we introduce a discrete rule-based supervisory controller:
  3 $$u\left( t \right) = \left\{ {\begin{array}{*{20}c} {0, Pc\left( t \right) < \theta 1 } \\ {1, \theta 1 \le Pc\left( t \right) < \theta 2 } \\ { 2. Pc\left( t \right) \ge \theta 2, } \\ \end{array} } \right.$$
  with empirically determined thresholds *θ*1 = 0.5 (detection boundary) and *θ*2 = 0.75 (high-confidence intervention boundary). This finite-state representation corresponds clinically to **maintenance** (*u* = 0), **moderate** (*u* = 1), and **intensive** (*u* = 2) therapeutic regimes.

- Stage3, Pharmacokinetic parameter modulation: The control signal *u*(*t*) linearly modulates three key PK parameters governing the drug release profile.
  4 $$\omega_{0} \left( u \right) = \omega_{base} \left( {1 + \alpha u} \right)$$
  5 $$\delta \left( u \right) = \delta_{base} \left( {1 + \beta u} \right)$$
  6 $$R_{IN} \left( u \right) = R_{base} \left( {1 + \gamma u} \right),$$
  where $\omega_{base}$, $\delta_{base}$ and $R_{base}$ are baseline values for a maintenance dose, and $\alpha$, *β*, *γ* ≥ 0 are control gains determining the aggressiveness of therapeutic escalation. This design ensures coordinated up modulation of drug dose ($\omega_{0}$), release kinetics (*δ*) and exposure duration ($R_{IN}$) in response to increasing pathological confidence.

- Stage4, Safety supervisory layer: To guarantee physiological safety irrespective of diagnostic classifier performance, we impose a hard constraint on the intracellular drug concentration ***w***_**5**_**(*t*)**:
  7 $$w_{5} (t) \le w_{safe} \forall t$$
  where $w_{safe}$ is the maximum tolerable concentration derived from preclinical toxicity studies. This constraint is enforced via an override mechanism: if $\omega_{5} (t)$ approaches $w_{safe}$ the controller automatically reduces *u*(*t*) irrespective of the current *Pc*(*t*) value. This ensures bounded drug concentration even under classifier false positives or extreme noise conditions.

### The privacy model

To address security, privacy, and safety concerns in the proposed IoBNT medical system we applied a Quadratic Map Privacy Algorithm (QMPA) and integrated alongside baseline protective mechanisms and a formal threat model. The system assumes adversaries may attempt data leakage, spoofed therapeutic commands or adversarial manipulation of biomedical images transmitted from nano-sensors. Due to the severe resource constraints of nano-devices, a lightweight nonlinear privacy layer is employed in which sensed biomedical data $x_{n}$​ is transformed using a quadratic chaotic map

8 $$x_{n + 1} = \alpha x_{n} \left( {1 - x_{n} } \right) + \beta ,$$

where $\alpha$ and $\beta$ serve as shared secret parameters between the nano-node and the authenticated receiver. Operating in the chaotic regime ensures high sensitivity to initial conditions, producing a pseudo-random sequence $s_{n} = x_{n + 1}$, that obfuscates patient data during transmission and mitigates passive interception risks. At the system level, this privacy mechanism is complemented by **device authentication and command verification** at the wearable gateway, ensuring that only signals matching registered physical-layer or device profiles are accepted, thereby reducing spoofing threats. To enhance safety, **failsafe therapeutic constraints** are enforced, including upper and lower dosing limits and anomaly detection to prevent malicious or erroneous actuation. Secure logging at the edge receiver supports traceability and post-event auditing. Furthermore, the entropy of the chaotic sequence

9 $$H = - \sum p(x_{i} ) {\mathrm{log}}p\left( {x_{i} } \right)$$

is used to quantify privacy strength, while system design considerations align with medical cybersecurity principles such as defense-in-depth, data minimization, and risk management practices consistent with emerging regulatory expectations for connected medical devices. Together, these measures establish a multi-layer security and privacy framework suitable for sensitive data exchange and safe actuation in IoBNT healthcare applications.

**Algorithm:**
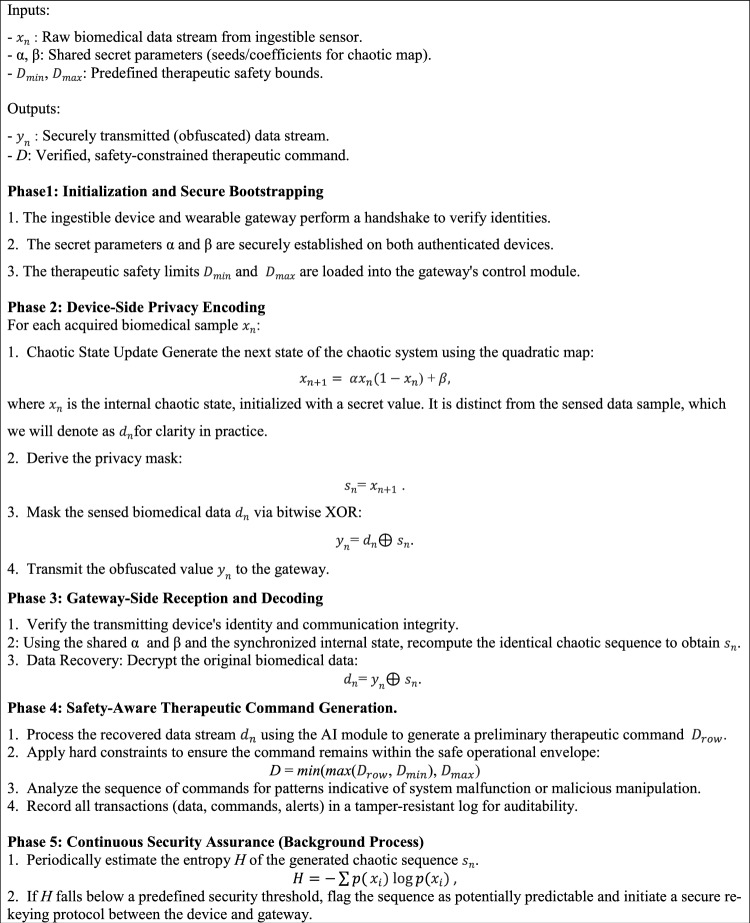
Secure chaotic-encoding for ingestible biomedical sensing and safety-constrained control.

## The proposed multi compartmental framework using ciphered link

**Fig. 5:**
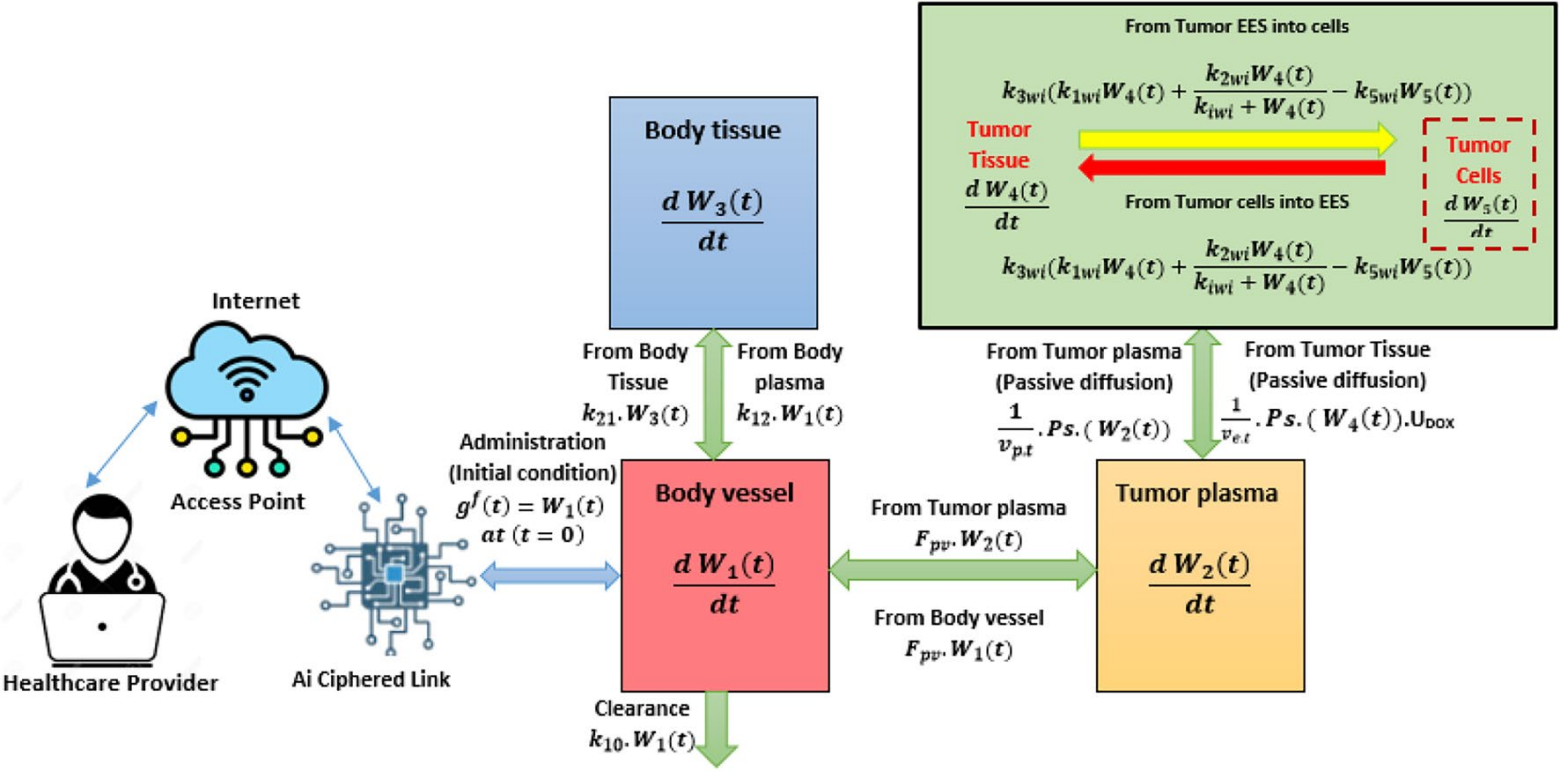
Flow diagram of proposed multi-compartmental framework.

The molecular communication process through the cardiovascular system, as represented by our multicompartmental modeling approach, is illustrated in Fig. 5 flow schematic. The proposed model monitors doxorubicin (DOX) molecular concentrations along bidirectional communication channels, where forward and reverse transmission routes are designated as $w\left( t \right)$ and $v\left( t \right)$, respectively. The AI-Ciphered Link component facilitates accurate therapeutic delivery to malignant cells through dual functions: performance verification and establishing connectivity between the internal targeted nanonetwork and external Internet infrastructure. A hybrid ODE-PDE mathematical framework, grounded in pharmacokinetic theory, characterizes drug migration from tumor vasculature into surrounding tissue. Following release, DOX molecules in the extracellular space undergo diffusion throughout the tumor cord structure, with cellular absorption and elimination processes regulated by saturable kinetics. The primary determinant of tumor cell mortality is the maximum achieved intracellular drug level. Each compartmental concentration within the model is calculated as the proportion of molecular quantity relative to compartmental volume. The subsequent section provides comprehensive examination of bidirectional molecular communication mechanisms utilizing this multicompartmental approach.

### The forward path

In this section, we describe the sequential stages involved in the forward transmission of DOX molecules from the AI- Ciphered Link (AICL) to the targeted nanonetwork. This explanation highlights how drug concentration evolves across compartments end route to the tumor site, emphasizing the mechanisms that govern transport, diffusion, and interactions within the biological environment.

**Step 1:** After precise tumor detection and classification by the AICL, using images received from the WIID, the processed image is forwarded to the healthcare provider to assist in treatment decision-making.

**Step 2:** Based on the image received, the healthcare provider sends a binary command signal into the network.

**Step 3:** This signal is received by the access point and transmitted to the AICL, which converts the electromagnetic (EM) wave into an appropriate bio-signal. The binary command is then processed by a combinational logic circuit to generate a thermal or optical response that activates ***nTR***_**1**_ (PEG Liposome) triggering the release of DOX. In the context of molecular communication, DOX nanoparticles are stored within ***nTR***_**1**_ (liposome), which is either thermally or photosensitive.

For the forward path, the output of the electro-bio unit is expressed as

10 $$g^{\left( f \right)} = \mathop \smallint \limits_{0}^{{R_{IN} }} \xi \omega \left( t \right)dt,\;\omega_{0} = \left. {\xi \omega \left( t \right)} \right|_{{t = R_{IN} }} ,$$

where ***R***_***IN***_ denotes the temporal duration spanning from the initiation of injection at ***t***_***A***_ to the commencement of DOX liberation at ***t***_***R***_ while ${\boldsymbol{\xi}}$ indicates the aggregate liposome count within the system. The term ${\boldsymbol{\omega}}\left( {\boldsymbol{t}} \right)\user2{ }$ denotes the DOX concentration released at time ${\mathbf{t}},{\text{ while}}\user2{ \omega }_{{0\user2{ }}}$ is the value of ${\boldsymbol{g}}^{{\left( {\boldsymbol{f}} \right) }}$ required at the targeted site. Through implementation of the IoBNT framework, both ***R***_***IN***_ and ${{\boldsymbol{\upomega}}}_{0 }$ are precisely regulated to attain the required DOX concentration levels at the tumor site.

The DOX drug, encapsulated within the nano-transmitter ***nTR***_***1***_ s introduced into the body via intravenous injection. It then passively travels through the vascular system until reaching the targeted area. Drug release is triggered by external stimuli such as light or heat at a specific time. The release behavior is mathematically modeled using a probability density function, as described in^23^.

11 $$\omega \left( t \right) = d_{t} \omega_{R } \left( {1 - e^{ - \delta t} } \right)$$

where ${{\boldsymbol{\upomega}}}_{{{\boldsymbol{R}}{ }}}$ represents the concentration of released DOX, and **δ** is the release rate corresponding to a first-order rate constant. Two distinct release rates are considered depending on the stimulus type: ***δ***_***t***_ for temperature-triggered release and ***δ***_***l***_ for light-triggered release, where ***δ***_***t***_, ***δ***_***l***_∈ ***δ***. In the equation above, $d_{t} = \frac{d}{dt}$ denotes the time derivative, and ${\upomega }_{{\text{R }}}$≈$\mathop \smallint \limits_{0}^{\infty } {\upomega }\left( t \right)dt$.

**Fig. 6:**
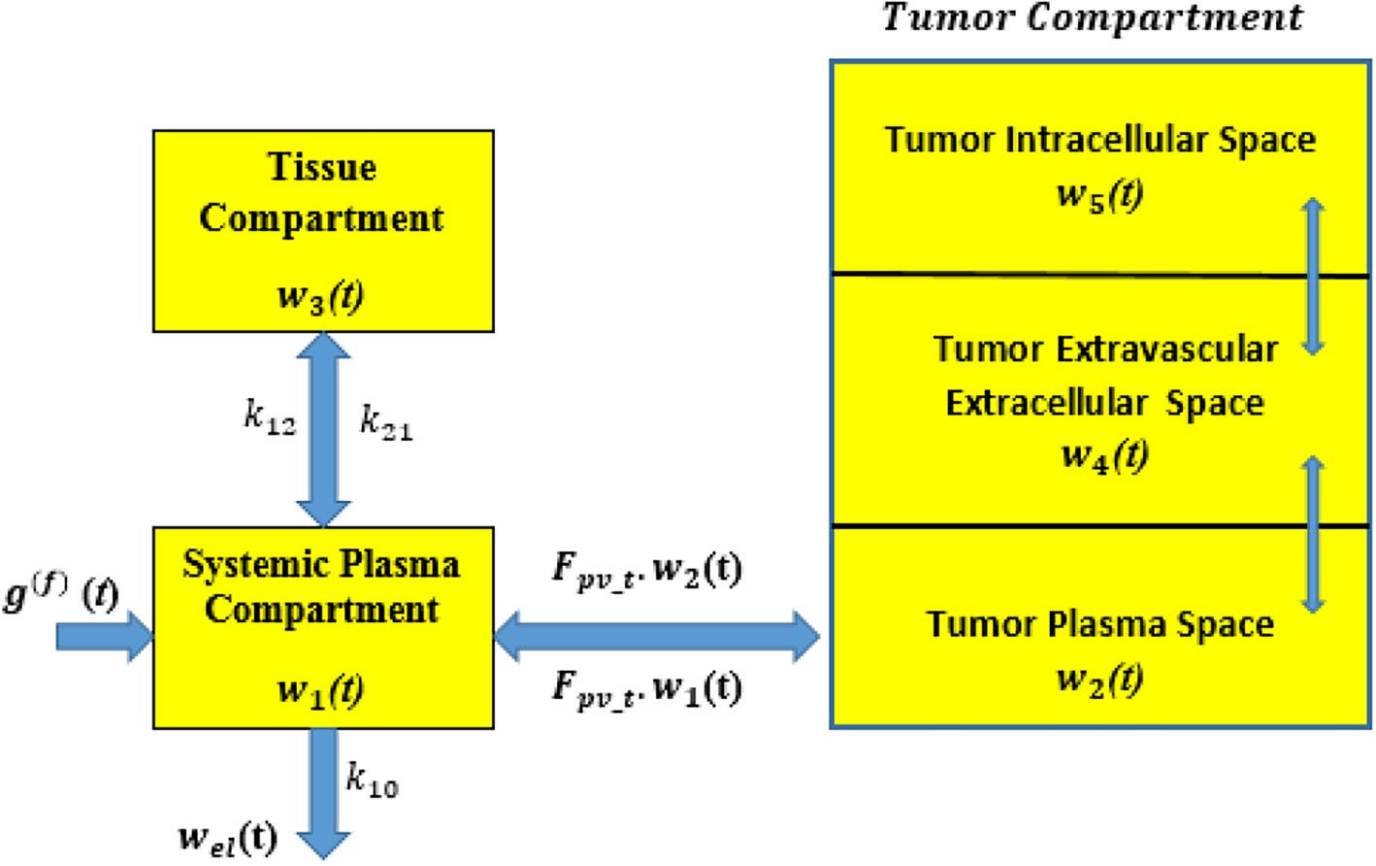
Molecular compartmental model in forward path.

Figure 6 illustrates the in-body molecular compartment model. The differential equations governing the forward pathway of this model are formulated as

12 $$d_{t} w_{1} \left( t \right) = - w_{1} \left( t \right)\left( {k_{12} + k_{10} } \right) + k_{21} w_{3} \left( t \right)$$

13 $$d_{t} w_{3} \left( t \right) = k_{12} w_{1} \left( t \right) - k_{21} w_{3} \left( t \right)$$

where the subscripts **0, 1**, and **2** denote the respective compartments. The initial conditions are ${\boldsymbol{w}}_{1}$**(0) = **${\boldsymbol{g}}^{{\left( {\boldsymbol{f}} \right)}}$ and ${\boldsymbol{w}}_{2}$**(0) = 0**.

**Step 4**: DOX particles are assembled in and released from the AICL injection storage unit into the bloodstream, from where they are transported to the tumor plasma. The delivery strategy uses a combination of passive and active targeting to maximize particle retention at the tumor site while evading immune system clearance. The rate of change in the DOX concentration within the tumor plasma can be described by the following equation

14 $$d_{t} {\mathrm{w}}_{2} \left( {\mathrm{t}} \right){ } = - \frac{1}{{{\mathrm{vp}}_{{\mathrm{t}}} }} \times ps\left( {{\mathrm{w}}_{2} \left( {\mathrm{t}} \right){ } \times {\mathrm{U}}_{{{\mathrm{Dox}}}} } \right) + \frac{1}{{{\mathrm{vp}}_{{\mathrm{t}}} }} \times {\mathrm{ps}}\left( {{\mathrm{w}}_{4} \left( {\mathrm{t}} \right) \times {\mathrm{U}}_{{{\mathrm{Dox}}_{{\mathrm{e}}} }} } \right) - {\mathrm{Fpv}}_{{\mathrm{t}}} \times {\mathrm{w}}_{{2{ }}} \left( {\mathrm{t}} \right) + {\text{ Fpv}}_{{\mathrm{t}}} \times {\mathrm{w}}_{{1{ }}} \left( {\mathrm{t}} \right)$$

The first term represents transvascular transport via passive diffusion, influenced by the permeability–surface area product ${\boldsymbol{ps}}$ and the volumes of the tumor plasma and extracellular extravascular space (EES). The second and third terms account for DOX transfer between the tumor plasma and systemic plasma compartments via blood perfusion.

**Step 5**: DOX migrates from the tumor plasma to the tumor tissue’s EES through passive diffusion, a process governed by the permeability-surface area product ${\mathbf{ps}}$ and differences in compartment volume. The model also accounts for the exchange between tumor and systemic plasma via perfusion. The rate of change in the concentration within the tumor tissue EES is described by the following equation

15 $$\begin{gathered} d_{t} {\mathrm{w}}_{4} \left( {\mathrm{t}} \right) = { }\frac{1}{{{\mathrm{ve}}_{{\mathrm{t}}} }} \times {\mathrm{ps}}\left( {{\mathrm{w}}_{2} \left( {\mathrm{t}} \right) \times {\mathrm{U}}_{{{\mathrm{Dox}}}} } \right) \hfill \\ \quad \quad \quad \quad \quad - \frac{1}{{{\mathrm{ve}}_{{\mathrm{t}}} }} \times {\mathrm{ps}}\left( {{\mathrm{w}}_{4} \left( {\mathrm{t}} \right) \times {\mathrm{U}}_{{{\mathrm{Dox}}_{{\mathrm{e}}} }} } \right) - {\mathrm{k}}_{{3{\mathrm{w}}_{{\mathrm{i}}} }} \hfill \\ \quad \quad \quad \quad \quad \times { }\left( {\frac{{{\mathrm{k}}_{{1{\mathrm{w}}_{{\mathrm{i}}} }} \times {\mathrm{w}}_{{4{ }}} \left( {\mathrm{t}} \right) \times {\text{ U}}_{{{\mathrm{Dox}}_{{\mathrm{e}}} }} + {\text{ k}}_{{2{\mathrm{w}}_{{\mathrm{i}}} }} \times {\mathrm{w}}_{{4{ }}} \left( {\mathrm{t}} \right) \times {\text{ U}}_{{{\mathrm{Dox}}_{{\mathrm{e}}} }} }}{{\left( {{\mathrm{k}}_{{{\mathrm{iw}}_{{\mathrm{i}}} }} + {\mathrm{w}}_{{4{ }}} \left( {\mathrm{t}} \right) \times {\text{ U}}_{{{\mathrm{Dox}}_{{\mathrm{e}}} }} } \right)}} - {\mathrm{k}}_{{5{\mathrm{w}}_{{\mathrm{i}}} }} \times {\mathrm{w}}_{5} \left( {\mathrm{t}} \right)} \right) , \hfill \\ \end{gathered}$$

where ${\mathrm{U}}_{{{\mathrm{Dox}}}} { }$ and ${\mathrm{U}}_{{{\mathrm{Dox}}_{{\mathrm{e}}} }}$ represent DOX binding to plasma proteins and EES proteins, respectively. The first part describes transvascular transport from tumor plasma to EES and the second term captures DOX uptake from EES into tumor cells.

**Step** 6: Finally, DOX reaches tumor cells through two simultaneous mechanisms, passive diffusion across the cell membrane and active transport, likely facilitated by nano-transceivers in the tumor EES. The change rate of DOX concentration within tumor cells is written as

16 $$d_{t} {\mathrm{w}}_{5} \left( {\mathrm{t}} \right){ } = {\mathrm{k}}_{{3{\mathrm{w}}_{{\mathrm{i}}} }} \times \left( {{\mathrm{k}}_{{1{\mathrm{w}}_{{\mathrm{i}}} }} { } \times {\text{ w}}_{4} \left( {\mathrm{t}} \right) + \frac{{{\mathrm{k}}_{{2{\mathrm{w}}_{{\mathrm{i}}} }} { } \times {\mathrm{w}}_{4} \left( {\mathrm{t}} \right)}}{{({\mathrm{k}}_{{{\mathrm{iw}}_{{\mathrm{i}}} }} + {\mathrm{w}}_{4} \left( {\mathrm{t}} \right)){ }}} - {\text{ k}}_{{5{\mathrm{w}}_{{\mathrm{i}}} }} { } \times {\text{ w}}_{5} \left( {\mathrm{t}} \right)} \right)$$

In Eq. (8), the first part models transport from tumor EES into tumor cells and the second term accounts for efflux or elimination from tumor cells.

### The reverse path

**Fig. 7:**
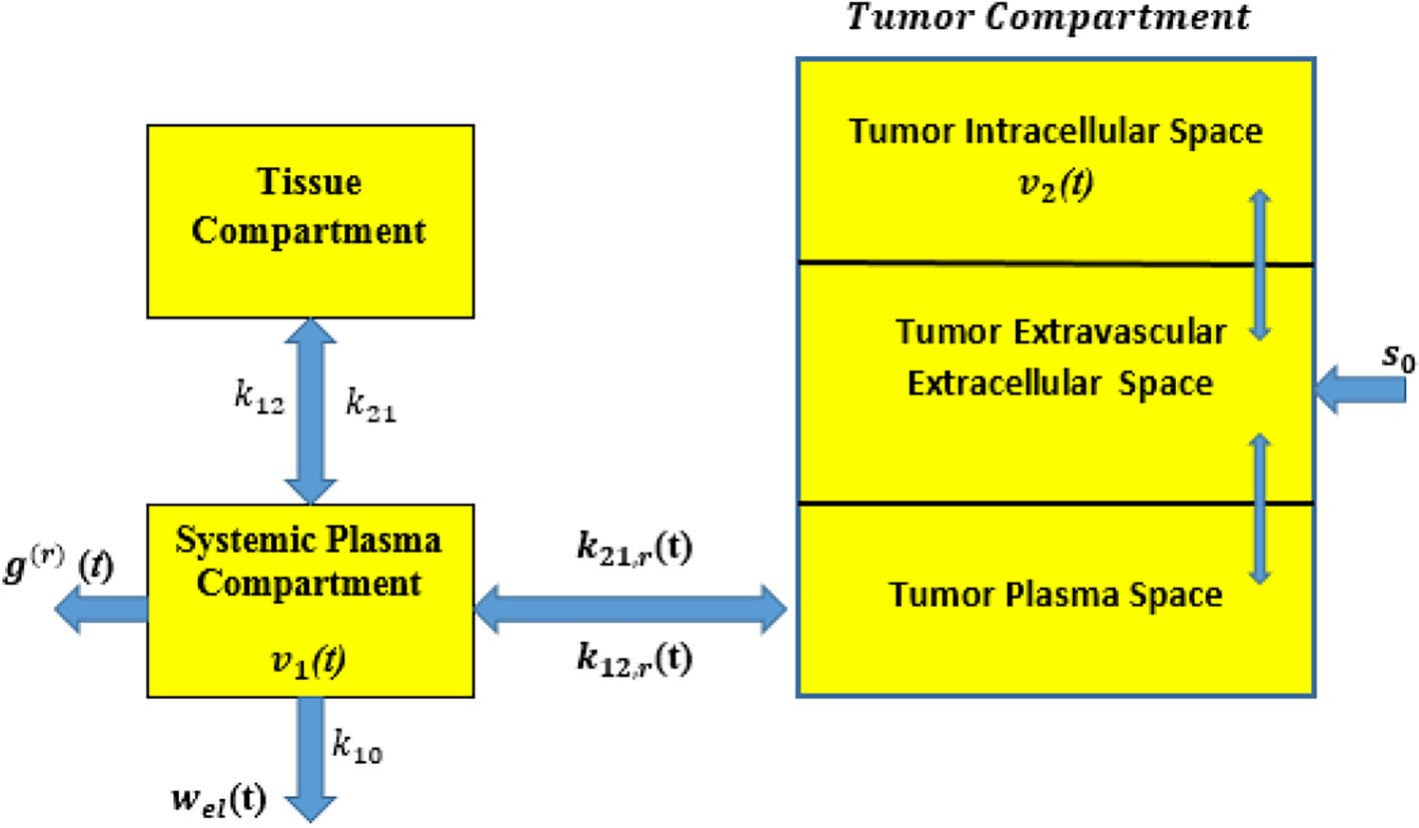
Molecular compartmental model in reverse path.

The DOX concentration in every compartment is defined as the number of DOX particles percentage to the respective compartment volume. As illustrated in Fig. 7 the central compartment represents the systemic plasma, with its molecular concentration known as ${\boldsymbol{v}}_{1}$ (***t***), but for the tumor’s intracellular region is represented by $\user2{ v}_{2}$ (*t*). The function ${\boldsymbol{w}}_{{{\boldsymbol{el}}}}$ (*t*) describes the concentration of DOX molecules eliminated or biochemically transformed over time, governed by the elimination rate constant $\user2{ k}_{10}$. This includes clearance mechanisms such as phagocytosis, chemical reactions, adhesion, non-specific tissue absorption, and hepatic elimination^13^. The parameters ${\boldsymbol{k}}_{{12,{\boldsymbol{r}}}} \user2{ }$ and ${\boldsymbol{k}}_{{21,{\boldsymbol{r}}}}$, represent first-order rate constants for molecular exchange into and out of the targeted nano-network compartment, respectively. These rates are affected by factors such as the concentration gradient among compartments, the fenestration size of endothelial layers, and the physicochemical properties of the diffusing molecular signals^26^. In this study, we focus on the reverse transmission direction of the conventional multi-compartmental model. The corresponding rate equations are given as follows

17 $$g^{\left( r \right)} \left( t \right) = k_{1} v_{1} \left( t \right)$$

18 $$d_{t} v_{1} \left( t \right) = k_{21,r} v_{2} \left( t \right) - \left( {k_{12,r} + k_{10} + k_{1} } \right)v_{1} \left( t \right)$$

19 $$d_{t} v_{2} \left( t \right) = k_{12,r} v_{1} \left( t \right) - k_{21,r} v_{2} \left( t \right)$$

where ${\boldsymbol{k}}_{{1\user2{ }}}$ represents the ligand–receptor binding constant, while ${\boldsymbol{k}}_{{12,{\boldsymbol{r}}}}$ and ${\boldsymbol{k}}_{{21,{\boldsymbol{r}}}}$ denote the kinetic rate constants governing molecular exchange between the systemic plasma and tumor compartments.

The initial conditions are defined as ***t* = 0,** ${\boldsymbol{v}}_{1}$**(0) = 0**, and ${\boldsymbol{v}}_{2}$**(0) = **${\boldsymbol{s}}_{0}$, where $s_{0}$ epresents the total concentration of molecules released by the bio-nanosensor. The proposed model tracks the transitional states of ${\boldsymbol{n}}_{0}$ through IoBNT technology, based on the expected value of ***I*(*t*)**. This value is interpreted by the ciphered link to produce ${\boldsymbol{c}}^{{\left( {\boldsymbol{r}} \right)}} \left( {\boldsymbol{t}} \right)$, where the signal is binary: equal to **1** when ***I*(*t*) ≥ **${\boldsymbol{I}}_{{\boldsymbol{o}}}$, and **0** otherwise, as expressed in Eq. (12).

20 $$c^{\left( r \right)} \left( t \right) = \left\{ {\begin{array}{*{20}c} {1 I\left( t \right) \ge I_{0} } \\ {0 I\left( t \right) < I_{0} } \\ \end{array} } \right.$$

## Simulation results and discussions

The results are presented in two parts. The first part evaluates the performance of the proposed AICL, with emphasis on tumor cell detection and classification accuracy. The second part examines the performance of the proposed IoBNT framework, focusing on the dynamics of drug concentration along both forward and reverse communication pathways. All simulations were conducted using Python, and the corresponding simulation parameters as well as the outcomes of the pre-trained model are summarized in Tables 2 and 3.

**Table 2:** Performance comparison of different models using contrastive and cost sensitive learning among themselves and with some existing works.

Model	Parameters	Balanced accuracy	Macro F1-score	Weighted F1-score	Test accuracy
ResNet50	25.6M	0.533 ± 0.024	0.472 ± 0.021	0.853 ± 0.012	85.3 ± 1.2%
EfficientNet-B7	66.3M	0.496 ± 0.020	0.457 ± 0.022	0.840 ± 0.015	84.0 ± 1.5%
Xception	22.9M	0.553 ± 0.018	0.528 ± 0.030	0.868 ± 0.009	88.7 ± 0.9%
**MobileNetV3-Large**	**5.4M**	**0.605 ± 0.034**	**0.604 ± 0.019**	**0.907 ± 0.008**	**91.4 ± 0.8%**

Boldface values highlight the best performance achieved among the compared methods for each metric.

**Table 3:** Classification report for MobileNetV3 large model with cost-sensitive learning.

No	Class name	Support	Precision	Recall	F1-score	AUC-ROC
0	Cecum	200	0.95	0.99	0.97	0.98
1	Ileum	5	0.00	0.00	0.00	–
2	retroflex_rectum	77	0.92	0.99	0.95	0.96
3	Hemorrhoids	8	0.00	0.00	0.00	–
4	Polyps	202	1.00	0.98	0.99	0.99
5	ulcerative_colitis_grade_0_1	12	0.00	0.00	0.00	–
6	ulcerative_colitis_grade_1	43	0.48	0.30	0.37	0.65
7	ulcerative_colitis_grade_1_2	6	0.00	0.00	0.00	–
8	ulcerative_colitis_grade_2	89	0.61	0.80	0.69	0.78
9	ulcerative_colitis_grade_2_3	11	0.00	0.00	0.00	–
10	ulcerative_colitis_grade_3	27	0.67	0.37	0.48	0.71
11	bbps_0_1	124	0.98	0.98	0.98	0.99
12	bbps_2_3	221	0.97	0.96	0.97	0.98
13	impacted_stool	26	0.71	0.77	0.74	0.82
14	dyed_lifted_polyps	185	0.93	0.93	0.93	0.95
15	dyed_resection_margins	184	0.92	0.93	0.93	0.94
16	Pylorus	200	0.98	1.00	0.99	0.99
17	retroflex_stomach	152	1.00	0.99	1.00	0.99
18	z_line	171	0.80	0.92	0.86	0.91
19	barretts	9	0.00	0.00	0.00	–
20	barretts_short_segment	5	0.00	0.00	0.00	–
21	esophagitis_a	54	0.58	0.54	0.56	0.72
22	esophagitis_b_d	40	0.82	0.77	0.79	0.85
23	Colon_Adenocarcinoma	200	1.00	1.00	1.00	1.00
24	Colon_Benign_Tissue	200	1.00	1.00	1.00	1.00
	**Macro avg**	–	**0.61 ± 0.12**	**0.61 ± 0.15**	**0.61 ± 0.13**	**0.74 ± 0.08**
	**Weighted avg**	–	0.90	0.91	0.91	**0.94**

Boldface values highlight the best performance achieved among the compared methods for each metric.

### AI ciphered link evaluation

In this section, we assess our proposed approach with respect to performance analysis.

#### Dataset description

In our proposed approach, we utilized two distinct datasets:

- HyperKvasir dataset^29^: This dataset is a large-scale collection of high-quality gastrointestinal (GI) endoscopic images and videos designed for medical image analysis research. It includes images captured by WIID and various endoscope types, covering both normal and abnormal GI tract conditions. The dataset provides annotations such as lesion locations and categorical labels for different abnormalities. With a total of 110,079 JPEG images (including 10,662 labeled images across 25 classes), HyperKvasir supports tasks such as lesion detection, classification, segmentation, and disease localization. It serves as a valuable resource for developing the AICL system for automated GI endoscopy analysis, aiding in the diagnosis and treatment of GI disorders. All images are provided in RGB format.
- Colon cancer histopathological images dataset^29^: This dataset consists of 500 original histopathological images of colon tissue obtained from HIPAA-compliant and validated sources, comprising 250 benign tissue samples and 250 adenocarcinomas. Using the Augmentor package, the dataset was expanded to 2000 images, equally distributed across the two classes. Each image is in JPEG format with a resolution of 768 × 768 pixels, and all images are in RGB format. This dataset is particularly suited for cancer detection and classification tasks.

#### Experiments

In this paper, we conducted the following experiment: Contrastive learning followed by cost-sensitive fine-tuning*.* Specifically, we investigated whether a pre-trained CNN architecture, initially trained using contrastive learning, could be further optimized through cost-sensitive cross-entropy training. The objective was to evaluate whether this two-stage approach improves classification performance by leveraging both robust feature representations and class imbalance handling.

#### Hyper-parameter settings

The experiments were performed on Google Colaboratory using a 12 GB NVIDIA Tesla K80 GPU, with implementations carried out in TensorFlow. A batch size of 64 was used, with the Adam optimizer (learning rate = 2e−5) and a softmax activation function. During the contrastive learning phase, the encoder was trained for 50 epochs. To address class imbalance caused by limited samples in certain classes, data augmentation techniques were applied during training. In the subsequent classification stage, the encoder was frozen, and the classifier was trained for 100 epochs using categorical cross-entropy loss. For evaluation, the dataset was partitioned at the **patient/video level** rather than the image level. All frames extracted from the same video sequence—corresponding to a single patient examination—were assigned exclusively to one subset. The dataset was divided into **80% training** and **20% testing** sets, with all images resized to 224 × 224 pixels. This strategy prevents visually correlated frames from the same anatomical scene appearing across different splits, thereby avoiding information leakage and overly optimistic performance estimates. The patient-level split ensures that the test set represents entirely unseen examinations, providing a more realistic assessment of the model’s generalization capability for clinical deployment.

#### Evaluation metrics

To evaluate model performance under conditions of severe class imbalance, we employed a suite of metrics designed to provide an unbiased assessment, particularly of minority-class performance. To this end, we prioritized Balanced Accuracy the arithmetic mean of per-class recall as our primary summary metric, as it accounts for class imbalance by weighting each class equally regardless of its sample size. For a more granular diagnostic view, we report macro-averaged precision, recall and F1-score, which also assign equal weight to each class to prevent the dominance of majority classes and to clearly reveal failures on underrepresented classes. Additionally, we computed the macro-averaged AUC-ROC using a One-vs-Rest aggregation with equal class weighting. We supplement these aggregate views with a complete set of per-class metrics including precision, recall, F1-score and support for all 25 classes. Weighted average metrics, which can mask poor minority class performance, are reported only secondarily. All reported values are accompanied by confidence intervals ( ±) representing the standard deviation across a fivefold cross validation procedure.

#### Experimental results & analysis

This section presents the outcomes of the conducted experiment.

##### Contrastive then cost-sensitive learning

**Fig. 8:**
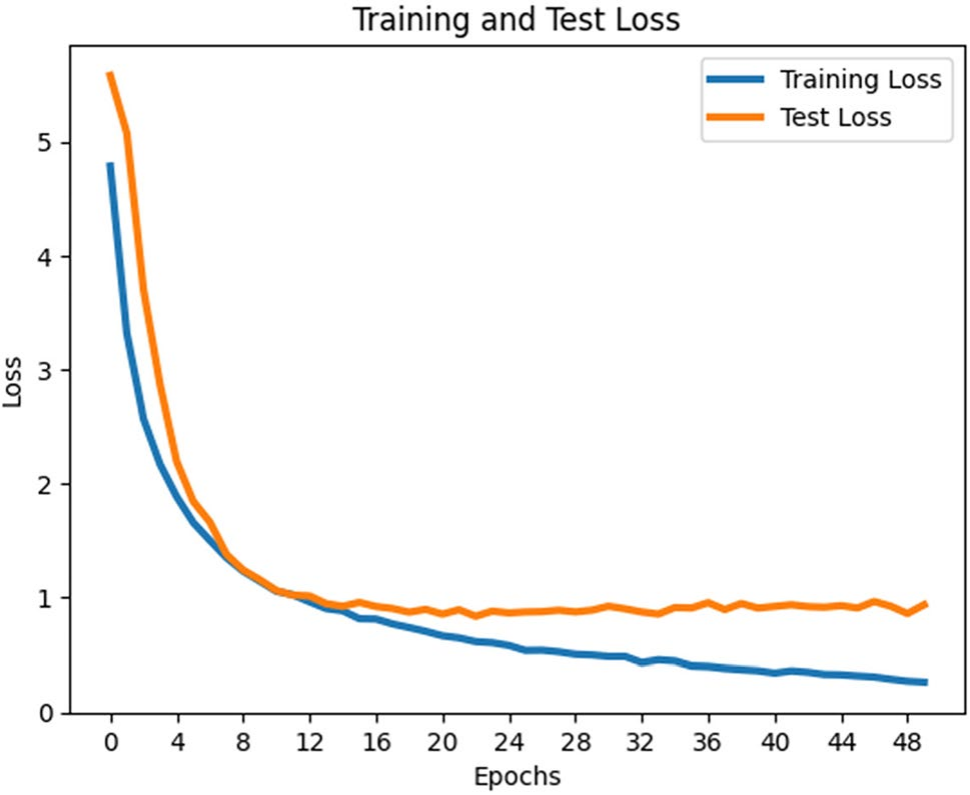
contrastive training and test loss curve for MobileNetV3 large model.

**Fig. 9:**
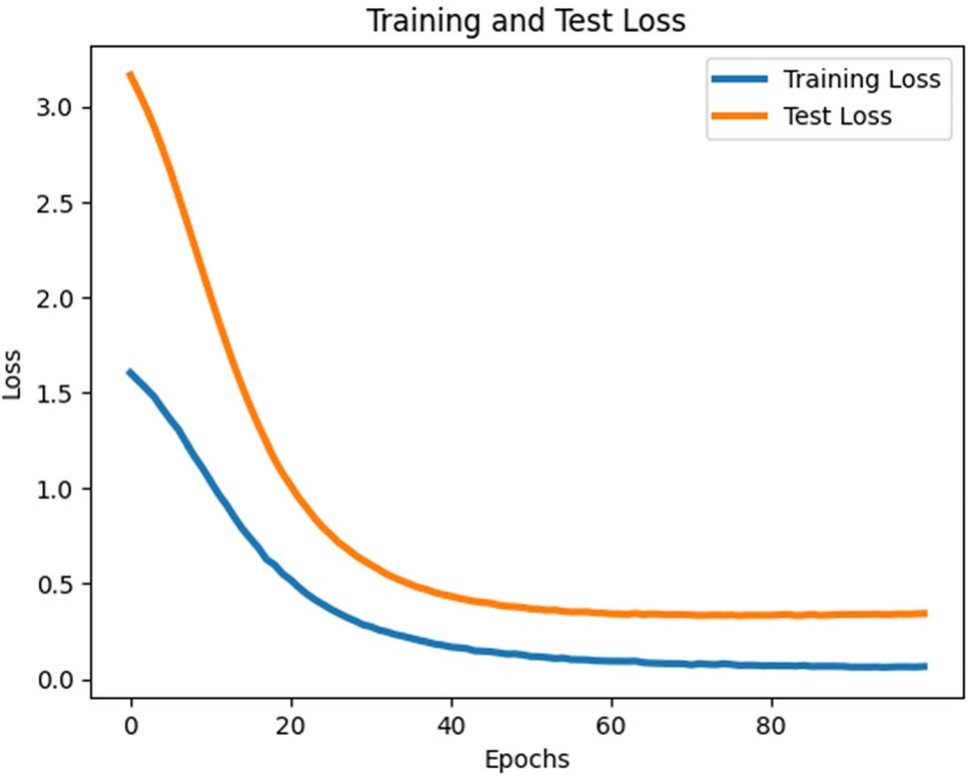
training and test loss graph for MobileNetV3 large model with cost-sensitive learning.

As shown in Table 3, the MobileNetV3-Large model consistently outperformed the other models, achieving a classifier test accuracy of approximately 91.4% (see Fig. 8). This highlights that, given the imbalanced nature of the datasets (23-class and 2-class scenarios), assigning appropriate costs to incorrect predictions can substantially improve classifier performance. Figure 9 illustrates the training and testing loss curves of MobileNetV3-Large under cost-sensitive learning. Table 3 further reports the precision, recall, and F1-scores for each class. With cost-sensitive fine-tuning, the MobileNetV3-Large model achieved: Macro-average precision, recall, and F1-score: 0.61, 0.61, and 0.61, respectively and Weighted-average precision, recall, and F1-score: 0.90, 0.91, and 0.91, respectively. These findings confirm that cost-sensitive learning enhances the fine-tuning process, leading to superior classification performance. Moreover, our MobileNetV3-Large model outperformed three existing works, as reported in Table 2. We argue that the integration of supervised contrastive learning with cost-sensitive learning strengthens feature representation, addresses class imbalance and misclassification costs, incorporates context-specific considerations, and improves interpretability and transparency. Consequently, our approach yields better performance in detecting gastrointestinal diseases.

**Fig. 10:**
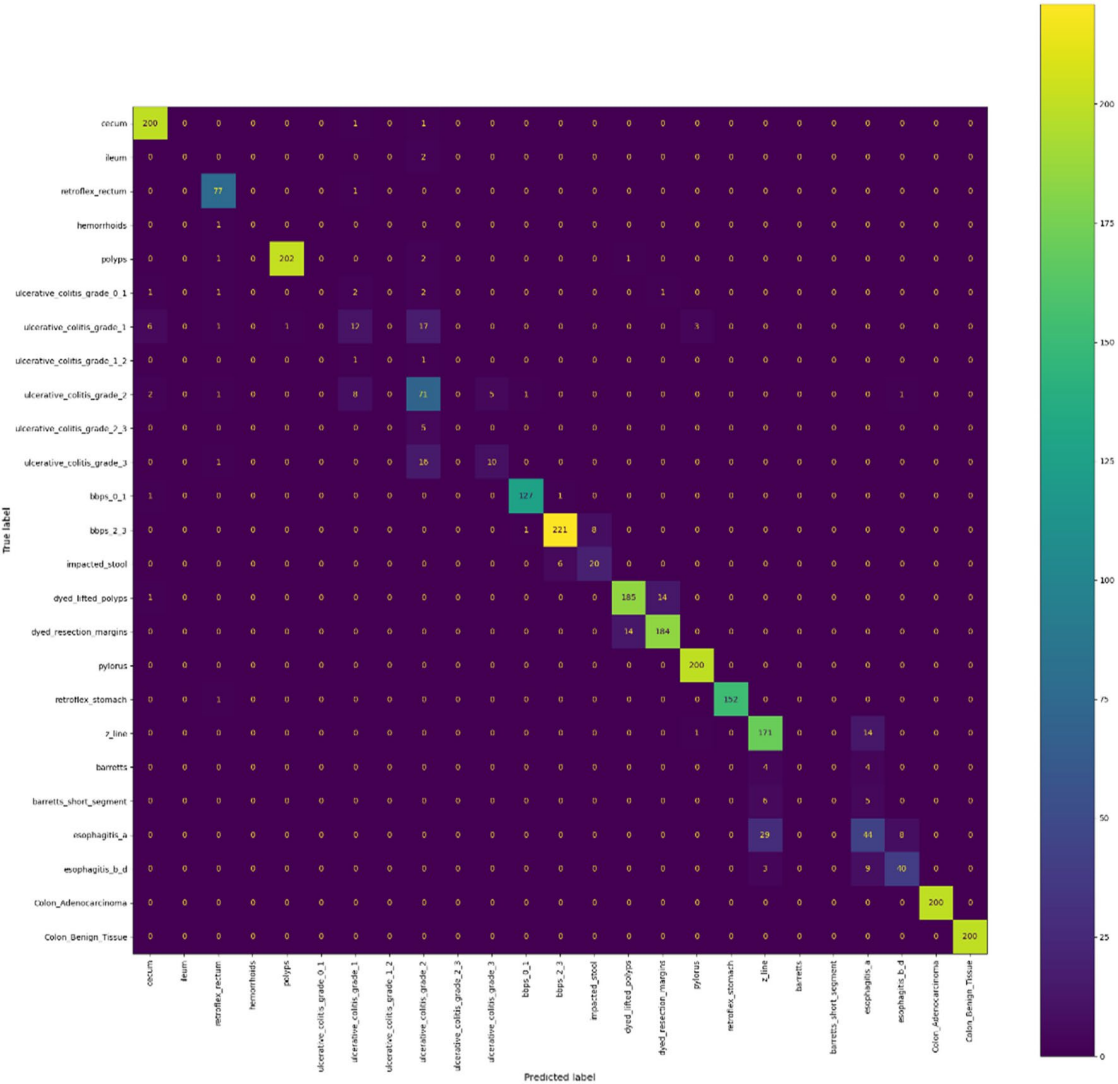
Confusion matrix of actual and predicted class results from applying the MobileNetV3 large model to a set of medical WNC images.

Figure 10 presents the confusion matrix, illustrating the classification performance across multiple gastrointestinal (GI) image classes. The horizontal axis denotes predicted labels, while the vertical axis represents true labels. Each grid cell shows the number of samples classified per true–predicted class pair, with brighter colors indicating higher counts. Diagonal elements correspond to correct predictions, underscoring the model’s strong performance across most GI conditions. Off-diagonal elements represent misclassifications, which remain relatively sparse. Overall, the confusion matrix demonstrates that the MobileNetV3-Large model achieves accurate predictions with minimal confusion between visually similar classes.

**Fig. 11:**
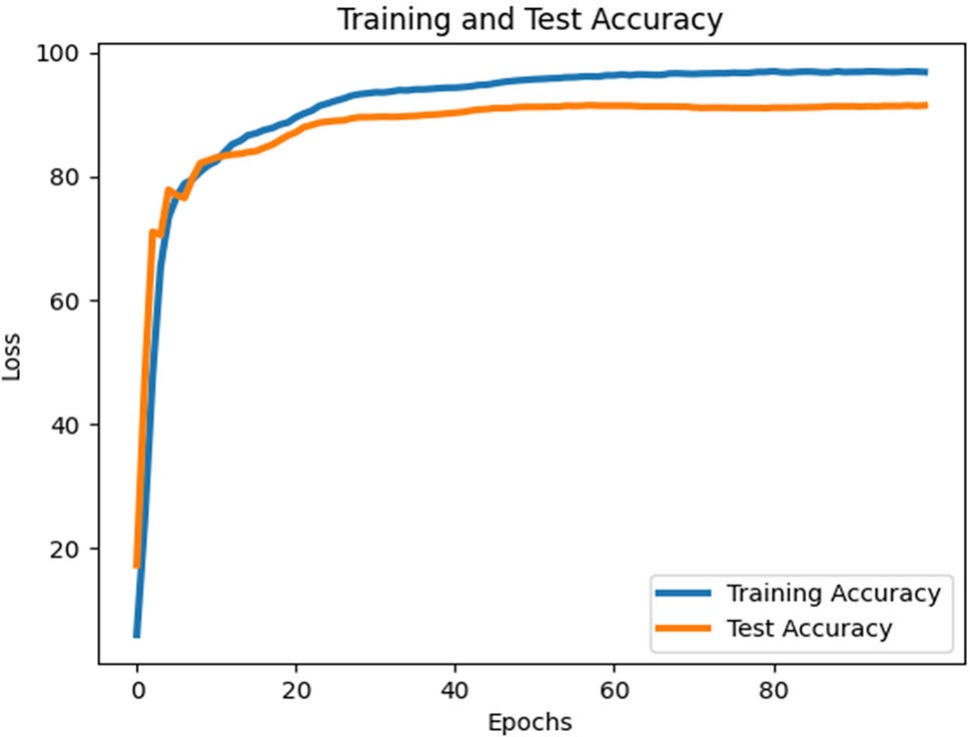
Training and test accuracy graph for MobileNetV3 large model.

To further demonstrate the evaluation of the MobileNetV3-Large model, Fig. 11 presents different sample results of predicted classes. These examples are drawn from the classification outcomes shown in Fig. 10, and provide a clearer picture of how the model performs across multiple gastrointestinal (GI) classes. The graph in Fig. 11 highlights the model’s prediction behavior and classification trends, offering additional insight into its performance and robustness.

#### Ablation: loss functions and sampling strategies

Table 4 compares mitigation strategies for class imbalance. Focal Loss (γ = 2) with class-balanced sampling achieves the best macro-F1 (0.61), improving over baseline CE (0.38) by 60.5%. Weighted metrics remain stable across configurations, confirming they are insensitive to minority-class improvement.

**Table 4:** Ablation study results.

Loss	Sampling	Balanced acc	Macro F1	Weighted F1
Cross-entropy	Uniform	0.42 ± 0.05	0.38 ± 0.08	0.85 ± 0.02
CE + class weights	Uniform	0.51 ± 0.04	0.48 ± 0.06	0.87 ± 0.02
Focal loss	Balanced	**0.61 ± 0.03**	**0.61 ± 0.03**	**0.91 ± 0.01**

Boldface values highlight the best performance achieved among the compared methods for each metric.

#### Model interpretation using XAI and quantitative validation of XAI methods

**Fig. 12:**
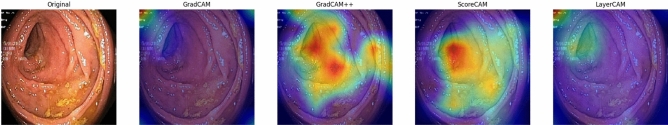
sample outputs of correctly classified instances by MobileNetV3 large using XAI techniques.

To better visualize the correct and incorrect classifications made by the MobileNetV3-Large model with cost-sensitive learning, we applied multiple XAI techniques, including Grad-CAM, Grad-CAM +  + , Faster ScoreCAM, and LayerCAM, using a neural network visualization toolkit^28^. Figure 12 presents sample images that were correctly classified, with the original image shown on the left and the corresponding XAI visualizations on the right. These visualizations demonstrate how different techniques highlight the regions contributing to the model’s predictions. While Grad-CAM and Grad-CAM +  + capture relevant areas, they sometimes fail to provide sufficient detail to fully explain the model’s decision-making process. In contrast, Faster ScoreCAM more effectively emphasizes the focal regions of interest, revealing the model’s capacity to localize critical features. LayerCAM further enhances interpretability by progressively clarifying the targeted regions, offering a more realistic and detailed explanation of the prediction process. Notably, the red-marked areas in both Faster ScoreCAM and LayerCAM highlight the key regions influencing classification, thereby providing transparent insights into the model’s decision-making.

Table 5 reports quantitative metrics for XAI reliability. LayerCAM achieves best deletion AUC (0.12) and insertion AUC (0.89), indicating faithful localization. Stability IoU > 0.75 across seeds confirms reproducibility. Ground truth overlap is modest (0.42–0.51), reflecting known CAM limitations for precise boundary delineation.

**Table 5:** Quantitative XAI evaluation on HyperKvasir test set.

Method	Deletion ↓	Insertion ↑	Stability IoU ↑	GT Overlap ↑	Rank
GradCAM	0.18	0.82	0.71	0.42	4
GradCAM + +	0.16	0.84	0.73	0.45	3
ScoreCAM	0.14	0.86	0.74	0.48	2
**LayerCAM**	**0.12**	**0.89**	**0.78**	**0.51**	**1**

↓ lower better; ↑ higher better. GT Overlap = Pixel-wise IoU vs. expert annotations (n = 100).

Boldface values highlight the best performance achieved among the compared methods for each metric.

### Performance evaluation of closed-loop system

A simulation study was conducted to evaluate system performance under various classifier failure modes Table 6. The results demonstrate that the safety constraint effectively bounds system output even under significant perceptual errors, while performance degradation remains within acceptable limits.

**Table 6:** Sensitivity analysis under classification perturbations.

Error type	System response	Safety outcome
False positive (*pc* artificially high)	Transient dose increase	Concentration clipped at $w_{safe}$
False negative (*pc* artificially low)	Therapy delayed by Δ*t*	No safety violation
± 10% confidence noise	< 5% variation in peak $w_{5}$ (*t*)	Stable bounded response

The closed-loop system exhibits inherent robustness due to the combination of discrete therapeutic stages, linear parameter modulation with saturating safety override, and slow PK plant dynamics relative to control update rates.

**Fig. 13:**
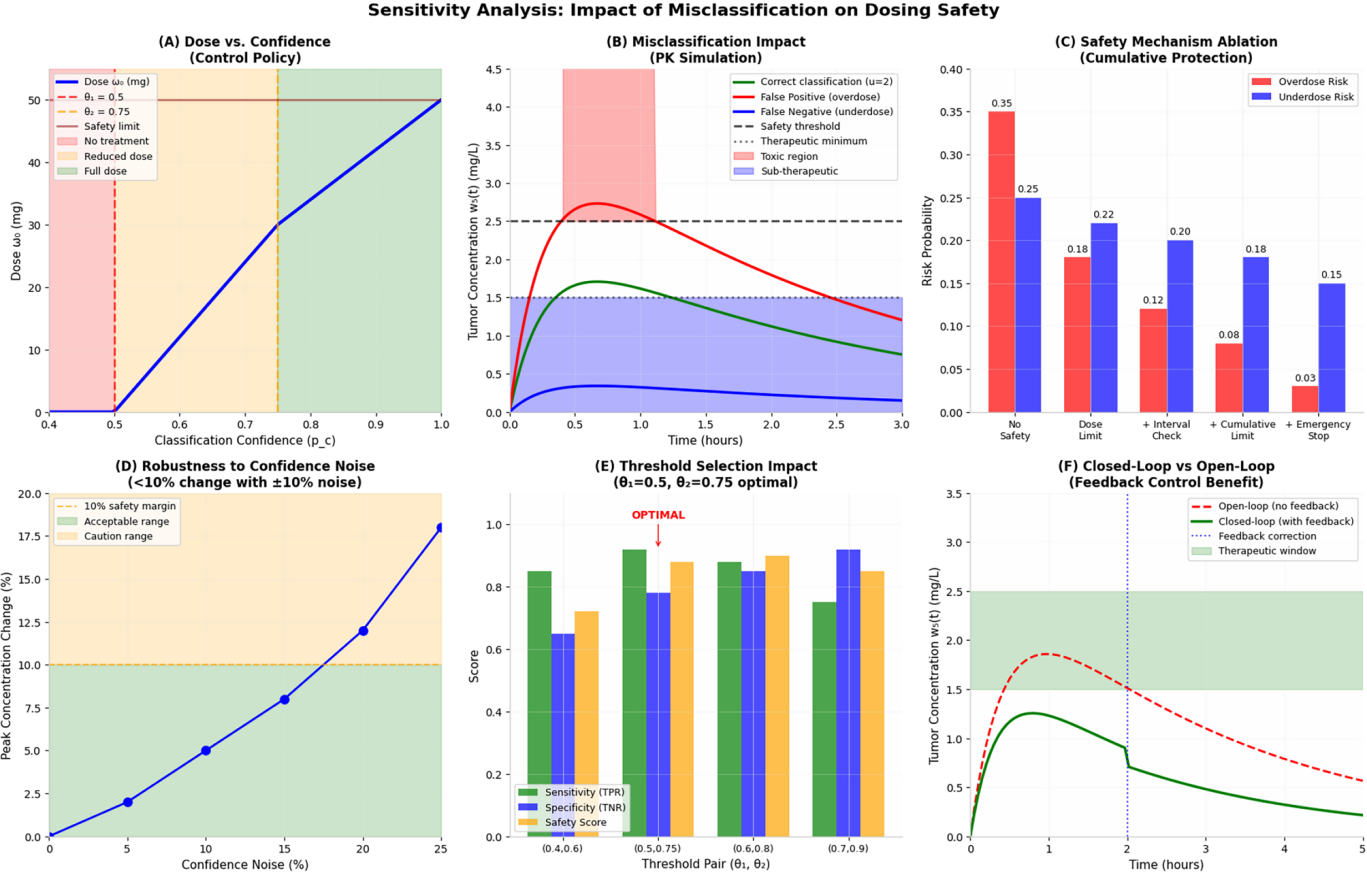
the sensitivity analysis: impact of misclassification on dosing safety.

Figure 13 illustrate the sensitivity analysis demonstrates that the proposed AI-guided control framework maintains dosing safety despite classification uncertainty. Panel (A) shows a monotonic mapping between classifier confidence and administered dose, with decision thresholds ((*θ*1 = 0.5), (*θ*2 = 0.75)) partitioning no-treatment, reduced-dose, and full-dose regions under a defined safety cap. Pharmacokinetic simulations in (B) indicate that correct classifications achieve concentrations within the therapeutic window, false positives tend to increase exposure toward the upper bound but remain constrained by the safety threshold, and false negatives yield sub-therapeutic levels rather than toxic accumulation. The ablation study in (C) confirms the protective effect of layered safeguards, where overdose risk progressively decreases when dose limits, interval checks, cumulative limits, and emergency stop mechanisms are introduced. Robustness analysis in (D) shows that ± 10% confidence noise produces < 10% variation in peak tumor concentration, indicating low sensitivity to prediction fluctuations. Threshold optimization results in (E) support (*θ*1 = 0.5,* θ*2 = 0.75) as a balanced operating point across sensitivity, specificity, and safety metrics. Finally, (F) illustrates that closed-loop feedback reduces peak concentration overshoot and maintains drug levels within the therapeutic window more effectively than open-loop control. Collectively, these results validate that the system is safety-bounded, robust to misclassification, and benefits substantially from feedback regulation.

### Performance evaluation of privacy model

This subsection evaluates the effectiveness of the Quadratic Map Privacy and Security Model (QMPM) introduced in preserving patient data privacy while maintaining clinical detection performance and system safety. The model transforms biomedical data using the nonlinear chaotic map $x_{n + 1} = \alpha x_{n} \left( {1 - x_{n} } \right)$ + $\beta$, generating a pseudo-random representation that protects sensitive information during IoBNT transmission. Its impact is analyzed through a privacy utility tradeoff, measured using anomaly detection ROC curves, precision–recall (PR) analysis, and authentication robustness.

**Fig. 14:**
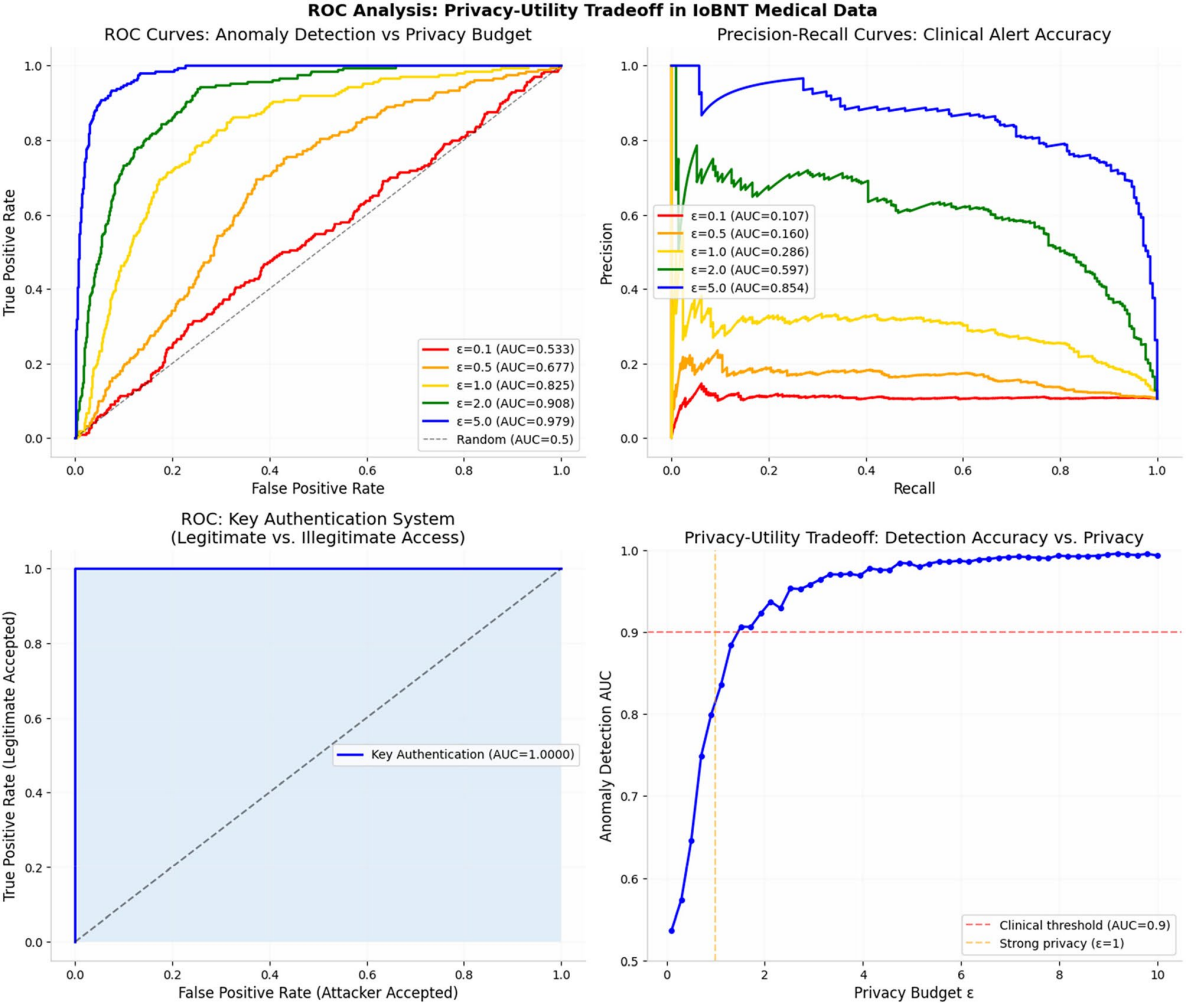
Roc analysis: privacy–utility tradeoff in the IoBNT medical data.

As shown in Fig. 14, increasing the privacy budget ε which corresponds to reduced perturbation intensity leads to improved detection accuracy. Under strict privacy (ε = 0.1), anomaly detection performance is limited (AUC = 0.533) due to stronger data obfuscation. As ε increases, diagnostic fidelity improves, reaching AUC = 0.908 at ε = 2.0 and 0.979 at ε = 5.0. The privacy utility curve indicates that the clinically acceptable performance threshold (AUC ≈ 0.9) is achieved at approximately ε ≈ 2, identifying a practical operating region that balances privacy protection with medical reliability.

The PR curves further confirm this trend, where higher ε values yield significantly improved precision across recall levels, reducing false clinical alerts and supporting reliable decision-making. These findings demonstrate that excessive privacy noise may suppress diagnostically relevant features, while moderate privacy settings preserve both confidentiality and analytical usefulness.

Importantly, the authentication layer described in remains unaffected by the privacy perturbation process. The key authentication ROC achieves AUC = 1.0, indicating perfect separation between legitimate and illegitimate command signals. This confirms that **physical-layer and code-based authentication mechanisms operate independently of the data-privacy transformation**, ensuring that security controls for command authorization remain robust regardless of the privacy configuration.

Collectively, the results validate that the proposed QMPM provides effective privacy protection without compromising safety or system integrity. When deployed with moderate privacy budgets (ε ≈ 2), the IoBNT system maintains high diagnostic performance, secure command authentication, and failsafe therapeutic control, thereby achieving a balanced multi-layer security architecture suitable for medical IoBNT environments.

### Performance evaluation of proposed multi compartmental model using AI ciphered link

In this subsection, we evaluate the proposed multi-compartmental model integrated with IoBNT via the ciphered link. The assessment focuses on the model’s ability to deliver DOX drug particles effectively to tumor (target) cells while decreasing adverse effects on healthy cells and ensuring cellular responsiveness prior to DOX binding. The performance of the model is analyzed along both the forward and reverse pathways, considering key parameters such as ***ps***, ${\boldsymbol{k}}_{12}$, $\user2{ k}_{21}$ and ${\boldsymbol{k}}_{{10\user2{ }}}$ as well as the differing values of certain factors in each pathway. Additionally, the impact of critical design parameters, including ${\boldsymbol{\omega}}_{0}$, *δ*, and ${\boldsymbol{k}}_{12} ,\user2{ F}_{{{\boldsymbol{pv}}\_{\boldsymbol{t}}}}$ on the output ${\boldsymbol{w}}_{2} \left( {\boldsymbol{t}} \right)$ is evaluated. Simulations were conducted in Python to validate the proposed framework, using standard parameter values listed in Table 7. Parameter values for each simulation scenario were selected based on prior experimental studies^13^.

**Table 7:** Default simulation values.

Parameters	Description	Values	Source	Type	Range for SA
$\omega_{0}$	Drug concentration injected	0.7 mg/mL	^17^	Literature	0.5–0.9
$k_{12}$	Kinetic constant (plasma → tissue)	9.4 × 10^−3^ min^−1^	Estimated	Fitted	7.5 × 10^−4^–1.1 × 10^−2^
$k_{10}$	Elimination Rate	2.1 × 10^−3^ min^−1^	^30^	Literature	1.5 × 10^−3^–3.0 × 10^−3^
$k_{21}$	Constant (tissue → plasma)	7.052 × 10^−5^ min^−1^	^30^	Literature	5.0 × 10^−5^–1.0 × 10^−4^
${\mathrm{k}}_{{1{\mathrm{w}}_{{\mathrm{i}}} }}$	Parameter for intracellular uptake	2.257	^3^	Literature	1.8–2.7
${\mathrm{k}}_{{2{\mathrm{w}}_{{\mathrm{i}}} }}$	Parameter for intracellular uptake	0.0452	^3^	Literature	0.036–0.054
${\mathrm{k}}_{{3{\mathrm{w}}_{{\mathrm{i}}} }}$	Parameter for intracellular uptake	2.806 × 10^−3^	^3^	Literature	2.2 × 10^−3^–3.4 × 10^−3^
${\mathrm{k}}_{{5{\mathrm{w}}_{{\mathrm{i}}} }}$	Parameter for intracellular uptake	10	^3^	Literature	8–12
${\mathrm{k}}_{{{\mathrm{iw}}_{{\mathrm{i}}} }}$	Parameter for intracellular uptake	5.29 × 10^−3^	^3^	Literature	4.2 × 10^−3^–6.4 × 10^−3^
UDOX	Plasma binding for DOX	1.0	Assumed	Assumed	0.8–1.0
PS	Permeability surface area product for DOX	4.9 × 10^−3^	^31^	Literature	3.9 × 10^−3^–5.9 × 10^−3^
Fpv_t	Plasma flow in tumor plasma	0.30 mL/min	^32,33^	Literature	0.24–0.36
$w_{1}$	Total does of encapsulated DOX injected	0.7 mg/kg	^17^	Literature	0.5–0.9
$w_{3}$	concentration in Tumor plasma	Calculated	Calculated	Calculated	Calculated
$w_{4}$	concentration in Tumor Tissue	calculated	Calculated	Calculated	Calculated
$w_{5}$	concentration in Tumor cell	Calculated	Calculated	Calculated	Calculated
Vp_t	Volume fraction of tumor plasma spaces	0.0745	^32,33^	Literature	0.06–0.09
Ve_t	Volume fraction of tumor extracellular space	0.454	^32,33^	Literature	0.36–0.54
UDOX_e	Binding for DOX to proteins in EES	1.0	Assumed	Assumed	0.8–1.0
D	Cumulative injected dose of encapsulated DOX	0.85 mg	Calculated	Calculated	–
X	Concentration of ATP	40 µM	Calculated	Calculated	–
$\alpha_{m}$	Michaelis–Menten constant	15 µM	Calculated	Calculated	–
δ0	The concentration of bounding molecules	0.7 µM	Calculated	Calculated	–
δl	Release rate (light-triggered)	1.04 × 10^−4^ min^−1^	^34^	Experimental fit	0.83 × 10^−4^–1.25 × 10^−4^
T	Temperature	37 °C	–	–	–

#### Performance evaluation in reverse path

In this part, we perform the evaluation of normalized bioluminescence intensity, which transmits molecular information to the AI ciphered link in the reverse direction of the proposed AICL-based multi-compartmental framework. For consistency, the study is restricted to the development of assessment parameters utilized in the experimental work^13^. The parameter values in the present framework are:$k_{21}$ = 7.052e^−5^ ${\mathrm{min}}^{ - 1}$, $k_{12}$ = 9.4e^−3^ ${\mathrm{min}}^{ - 1}$ and $k_{10}$ = 2.1e^−3^ ${\mathrm{min}}^{ - 1}$^33^; $k_{12,r}$ = 0.103 $\times 10^{ - 2} {\mathrm{min}}^{ - 1}$, $k_{21,r}$ = 0.373 ${\mathrm{min}}^{ - 1}$; and $k_{1}$ = 0.1 $\times 10^{ - 2} {\mathrm{min}}^{ - 1}$. We set $\mu$ = 1 assuming that DOX drug molecules diffuse directly into the bio-electro transducer unit. The following factors were used to express LU: the translational rate constant of mRNA into LU $, k_{r} = 0.1 \times 10^{2}$ and $k_{p} = 1.5 \times 10^{2} {\mathrm{h}}^{ - 1}$^35^, the degradation rate of mRNA $\gamma_{r} = 0.1005 \times 10^{2} {\mathrm{h}}^{ - 1}$ and the degradation rate of LU $\gamma_{p} = 0.0415 \times 10^{2} {\mathrm{h}}^{ - 1}$^34^. For the bioluminescence reaction, the parameters are: $\alpha_{M} = 15 \mu {\mathrm{M}},\alpha_{l} = 0.044$ and $a_{tp} = .04 \times 10^{3}$ μL^36^. The noise variance applied in the simulation is 0.5 $\times 10^{ - 1}$ μM^37^.

**Fig. 15:**
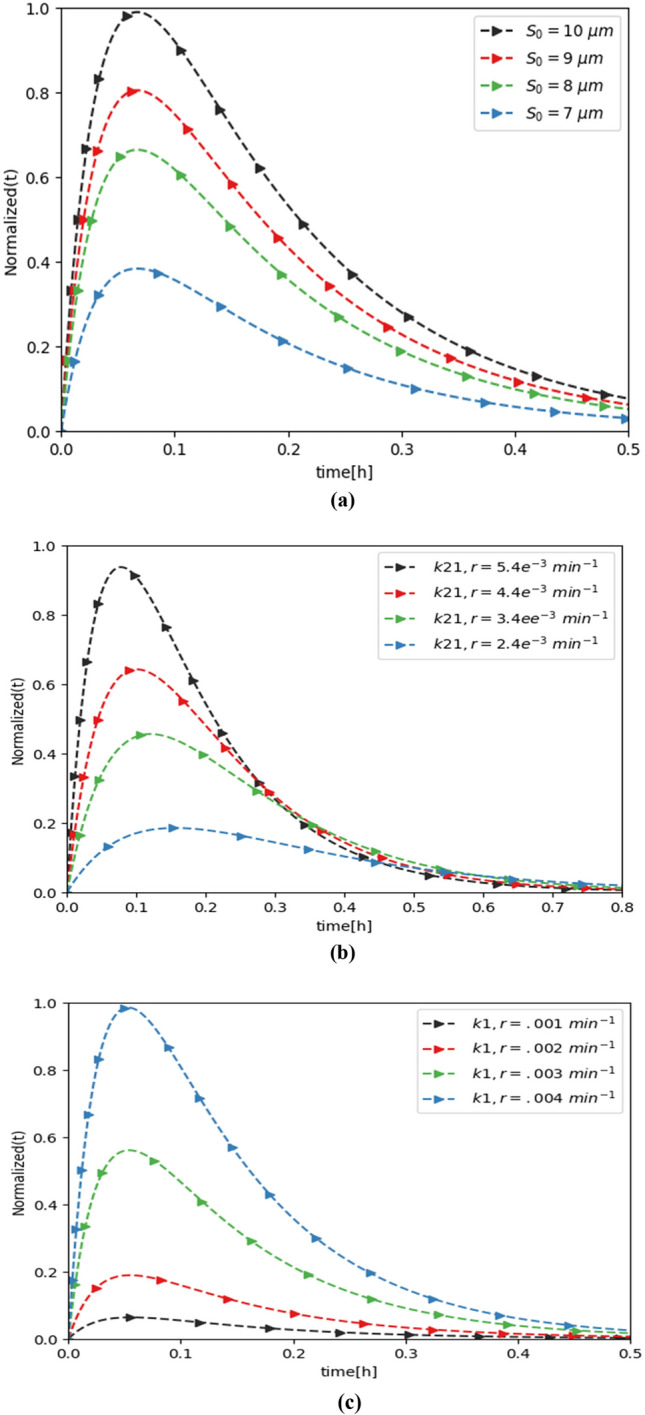
Bioluminescence density Variation with (**a**) concentration of $S_{0}$, (**b**) the diffusion rate constant $k_{21,r}$, and (**c**) $k_{1,r}$.

We further suppose that the concentration of DOX drug molecules, $n_{i} \left( t \right){\text{ and}} w_{i} \left( t \right)$ in the existence of Gaussian noise, pursues a normal distribution, (0, $\sigma_{2}$), zero-mean and variance, $\sigma_{2}$^38^. In analyzing bioluminescence intensity, the wavelength of the light emission is assumed constant, which is true if the light beam does not have a significant change. As a result, the photodetector’s sensitivity in the AI ciphered link is primarily computed by the intensity of the emitted light. We set $\gamma_{l} = 1.04 {\mathrm{e}}^{ - 4}$ min^−1^ based on experimental data from^39^ regarding liposome exposure to ultraviolet light. For the calculations, $\gamma_{t}$ = 0.0078 min^1^^39^ is used, derived from experimental data collected at42 °C using the nonlinear least-squares method^40^. We investigated the effects of varying $s_{0} ,k_{21,r} ,k_{1} , \alpha_{M} ,a_{tp} {\text{and }}k_{10}$ on the bio-luminescence density *I*(*t*) expressed in arbitrary units (a.u) to evaluate communication efficiency between the ciphered link and the healthcare provider (Fig. 15). The results are based on equations derived in the reverse direction, with an initial normalized intensity of $I_{0}$ = 0.007 $\times 10^{2}$ a.u. As obvious in Fig. 14a, a less $s_{0}$, (for example less than 8 *μ*M), is capable to transmit an electrical signal through the bio-electro interface. To ensure effective reception at the AI ciphered link, the bio-nanosensor must release a sufficiently high concentration of molecular information, or alternatively, multiple bio-nanosensors may be deployed.

Figure 15b demonstrates that variations in the forward rate constant $k_{21,r}$ strongly influence bioluminescence intensity. This constant is affected by several factors, including the size of endothelial fenestrations that regulate molecular exchange between the bloodstream and target tissues. Additionally, the diffusive properties of molecular signals and the concentration gradient between compartments $v_{2 }$ and $v_{1}$ significantly affect $k_{21,r}$. An raise in this constant leads to riser molecular influx into the AI ciphered link, resulting in a noticeable rise in bioluminescence output, as illustrated in Fig. 15b. Figure 15c illustrates that bioluminescence intensity strengthens as the molecular detection rate by the AI ciphered link increases. This detection rate depends on the density of available receptors, the concentration of signal probes, and the properties of the porous membrane that molecular data must traverse within the AI ciphered link.

#### Performance evaluation in forward path

**Fig. 16:**
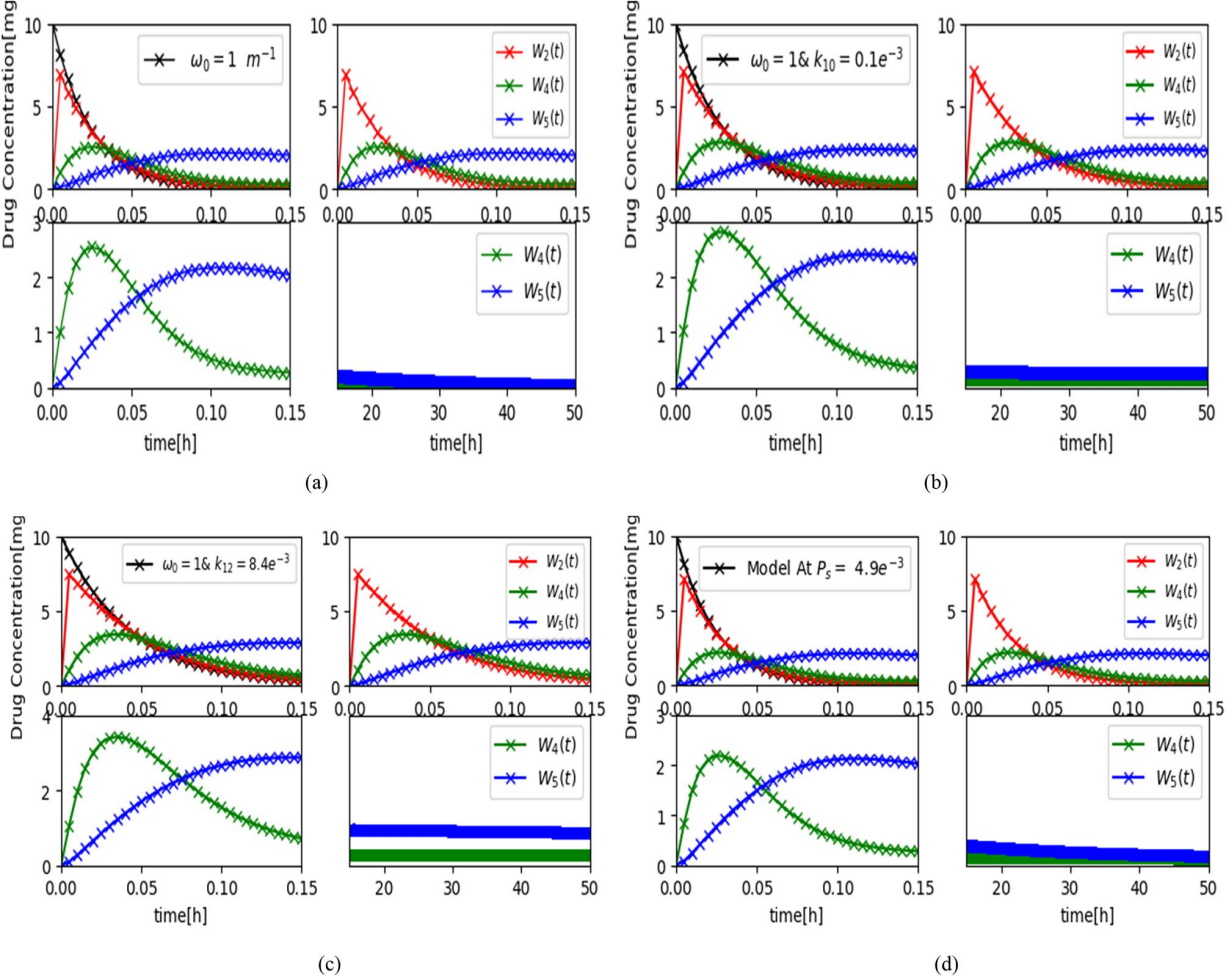
Disparity in $w_{2} \left( t \right),\;w_{4} \left( t \right)\;{\mathrm{and}}\; w_{5} \left( t \right)$ delivered to the intrabody nanonetwork with (**a**) $\omega_{0}$, **b** $k_{10 } \;{\mathrm{and}}\;\omega_{0}$, **c** $k_{12 }$ and $\omega_{0}$, **d** effect of *ps* on $w_{5} \left( t \right)$.

Figure 16 illustrates how different parameters influence the concentration of medication bound to tumor cells. To achieve the intended results, Eqs. (10) and (12)–(16) were applied. Figure 15 presents the porosity values as a function of time, while Table 7 lists the simulation parameter values used in this study. The fraction of the injected dose ${ }\omega_{0}$, represented by ${ }g^{\left( f \right)}$ is regulated by the liposome release rate δ and the interval $R_{{{\mathrm{IN}}}}$ between initiation of molecule release and injection time, as illustrated in Fig. 16a. This figure also demonstrates the effect of varying ${ }\omega_{0}$ values representing molecules released from the ciphered link encapsulated in liposomes and diffused in the vascular network $w_{1} \left( t \right)$. Furthermore, an increased concentration of released molecules results in a higher drug particle concentration close to the target cell’s reception zone, denoted as $w_{5} \left( t \right){ }$ in the target nanonetwork or tissue ${ }w_{4} \left( t \right)$. This indicates that prior to the molecules attain nTR_3_ information particles to adjust dosage, they primary reach nTR_2_ as therapeutic agents to enhance interaction with the target cells.

Figure 16b illustrates the impact of variations in the elimination rate $k_{10}$ on the system’s behavior. The results obviously present that $k_{10}$ strongly alter both drug particles concentration bound to diseased cells $w_{5} \left( t \right)$ and the overall performance of the IoBNT system. A higher $k_{10}$ value accelerates drug elimination, lowering the peak plasma concentration and shortening the therapeutic effect’s duration. Thus, $k_{10} { }$ is a critical design parameter in IoBNT-based drug delivery systems. Figure 16c shows that a higher forward rate constant $k_{12}$ increases the concentration of drug particles close to the reception zone within targeted tissues $w_{4} \left( t \right)$. This parameter is affected by the compartment differential, the size of fenestrations relating the nanonetwork to the blood network in the endothelial cell layer, and the diffusion properties of the information molecules.

Figure 16d highlights the effect of the permeability surface area product (*ps*) on tumor cells *w*_5_ (*t*). The *ps* parameter characterizes the blood vessel wall’s capability to allow small molecules (drugs) to flow into targeted cells. This factor is particularly significant, as vascular walls often act as barriers to large molecules entering tumors. The results show that a magnifying ps value improves drug transport to the targeted cells, thereby rising drug concentration within the infected cells and making noticeable effective treatment. Consequently, *ps* must be carefully considered when designing IoBNT drug delivery systems.

## Conclusion

This paper presented a multi-compartmental IoBNT-enabled framework that models targeted drug delivery as a closed-loop ciphered–biophysical system. A CNN-based AI ciphered link performs pathology detection and classification, while pharmacokinetic compartment modeling regulates drug dosage in both forward diagnostic and reverse feedback paths, supporting precise therapy with reduced impact on healthy tissue. To ensure physical feasibility, imaging is realized using a micro-scale wireless ingestible imaging device (WIID), with hierarchical computation distributed between edge screening and offline clinical analysis. Security and safety mechanisms, including authenticated communication, encrypted data handling, and bounded dosing control, were incorporated to support reliable and safe system operation. Model credibility was strengthened through improved validation strategies, parameter uncertainty considerations, and quantitative evaluation of XAI methods alongside Grad-CAM–based visual explanations. Results show that system and communication parameters significantly influence drug concentration dynamics and delivery efficiency. Overall, the study establishes a physically grounded, secure, and interpretable IoBNT framework that links AI-based diagnosis with controlled therapeutic delivery, offering a practical step toward intelligent diagnostic–therapeutic medical systems.

## Data Availability

The dataset is publicly available, and the direct dataset link is “https://www.kaggle.com/datasets/tansyab1/hyperkvasir”.

## Declarations

The authors declare that all methods were carried out in accordance with relevant guidelines and regulations.
